# Fluorescence fluctuation‐based super‐resolution microscopy: Basic concepts for an easy start

**DOI:** 10.1111/jmi.13135

**Published:** 2022-08-09

**Authors:** Alma Alva, Eduardo Brito‐Alarcón, Alejandro Linares, Esley Torres‐García, Haydee O. Hernández, Raúl Pinto‐Cámara, Damián Martínez, Paul Hernández‐Herrera, Rocco D'Antuono, Christopher Wood, Adán Guerrero

**Affiliations:** ^1^ Laboratorio Nacional de Microscopía Avanzada, Instituto de Biotecnología Universidad Nacional Autónoma de México Cuernavaca Morelos Mexico; ^2^ Centro de Investigación en Ciencias, Instituto de Investigación en Ciencias Básicas y Aplicadas Universidad Autónoma del Estado de Morelos Cuernavaca Morelos Mexico; ^3^ Posgrado en Ciencia e Ingeniería de la Computación Universidad Nacional Autónoma de México Mexico City Mexico; ^4^ Crick Advanced Light Microscopy Science and Technology Platform The Francis Crick Institute London UK

**Keywords:** super‐resolution microscopy, fluorescence fluctuation, bioimaging, computational biology, fluorescence microscopy, nanoscopy

## Abstract

Due to the wave nature of light, optical microscopy has a lower‐bound lateral resolution limit of approximately half of the wavelength of visible light, that is, within the range of 200 to 350 nm. Fluorescence fluctuation‐based super‐resolution microscopy (FF‐SRM) is a term used to encompass a collection of image analysis techniques that rely on the statistical processing of temporal variations of the fluorescence signal. FF‐SRM aims to reduce the uncertainty of the location of fluorophores within an image, often improving spatial resolution by several tens of nanometers. FF‐SRM is suitable for live‐cell imaging due to its compatibility with most fluorescent probes and relatively simple instrumental and experimental requirements, which are mostly camera‐based epifluorescence instruments. Each FF‐SRM approach has strengths and weaknesses, which depend directly on the underlying statistical principles through which enhanced spatial resolution is achieved. In this review, the basic concepts and principles behind a range of FF‐SRM methods published to date are described. Their operational parameters are explained and guidance for their selection is provided.

## Lay description

Due to light's wave nature, an optical microscope's resolution range is 200 to 350 nanometers. Several techniques enhance resolution; this work encompasses several fluorescence fluctuation super‐resolution (FF‐SMR) methods capable of achieving nanoscopic scales. FF‐SRM is known to be suitable for fixed or live‐cell imaging and compatible with most conventional microscope setups found in a laboratory. However, each FF‐SRM approach has its strengths and weaknesses, which depend directly on the underlying principles through which enhanced spatial resolution is achieved. Therefore, the basic concepts and principles behind diverse FF‐SRM methods are revisited in this review. In addition, their operational parameters are explained, and guidance for their selection is provided for microscopists interested in FF‐SRM.

## 1 INTRODUCTION

### 1.1 Basic concepts of fluorescence and the resolution limit of optical microscopy

Fluorescence microscopy, in which organic and inorganic fluorophores are used as molecular dyes, is by far the most popular technique for the observation of biological specimens with molecular specificity.^1^ This level of discrimination is achieved either by labeling cellular components with fluorescent dyes or by linking an engineered fluorescent protein to a molecule of interest.^2^

**FIGURE 1:**
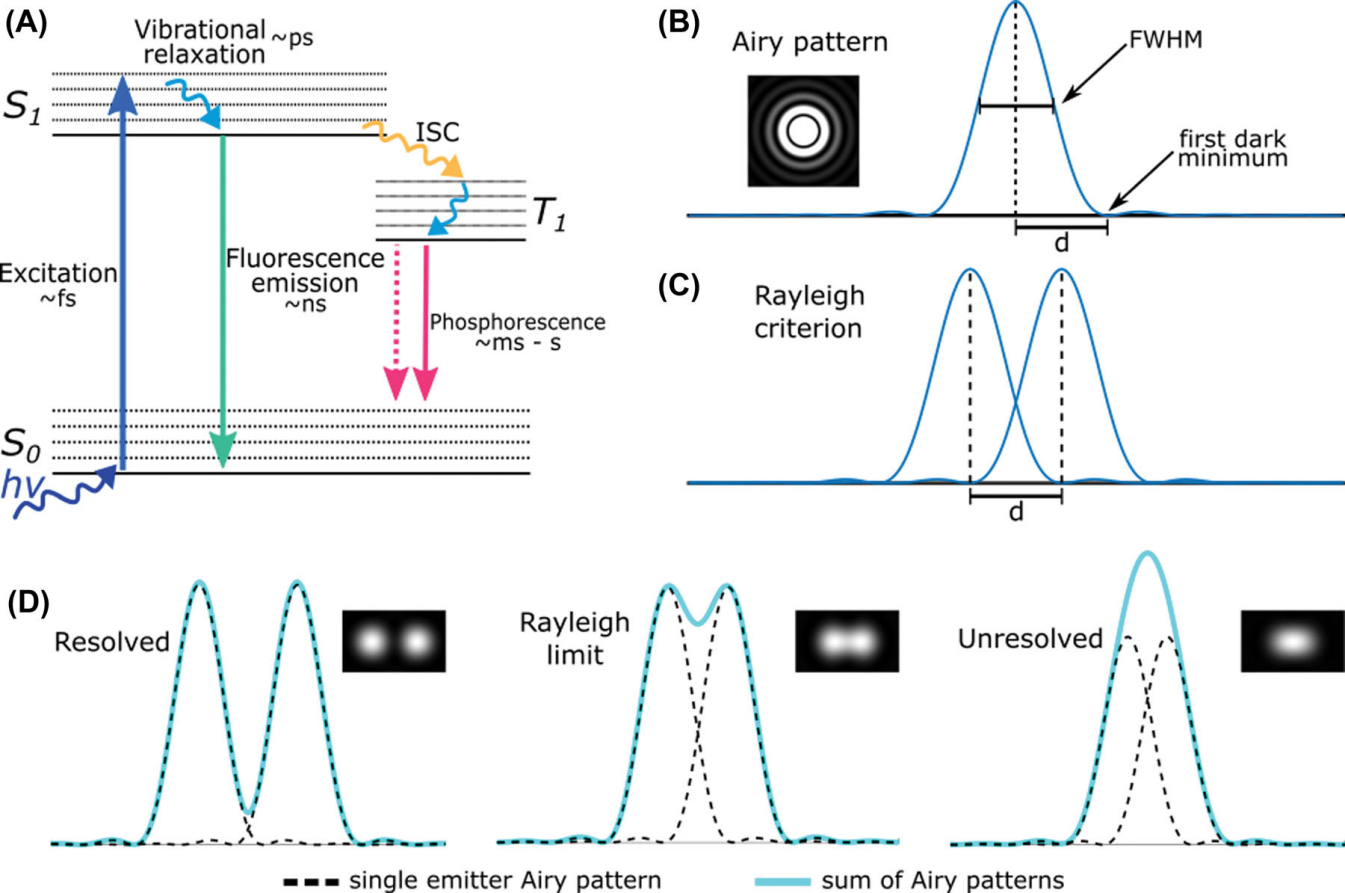
Fluorescence process described by the Jablonski diagram and the spatial resolution limit according to the Rayleigh criterion. (A) Simplified Jablonski diagram representing energy states of a fluorescent molecule and their characteristic timescales. The excitation photon transfers energy *h*ν to the fluorophore that promotes the transition of an electron from the ground state *S*_0_ to the excited state *S*_1_. The excited electrons can return to *S*_0_ through radiative emission of a photon (fluorescence) or by a non‐radiative transition. Less frequently, the electrons can reach a triplet state (*T*_1_) by intersystem crossing (ISC) upon which a change occurs to the spin of the electron. (B) Bidimensional representation of the point spread function (PSF) formed by the Airy pattern of one emitter and (C) the corresponding Rayleigh criterion for optical resolution when two of these patterns are in close enough proximity to each other. (D) Fluorescence distributions of two fluorescence emitters separated at three different distances. From left to right, the emitters are spatially resolved, at the limit of resolution (according to the Rayleigh criterion) or unresolved. ‘d’ refers to the distance between two points. FWHM, full width at half maximum

The Jablonski diagram represents the electronic transitions between energy states during the excitation and relaxation of a fluorophore (Figure 1A). Fluorescence occurs when a fluorophore in a ground electronic state (*S*_0_) absorbs photonic energy at a specific wavelength range, which promotes an electron to shift to a higher‐energy excited state (*S_n_*). Energy is released upon return of the electron to *S*_0_, either by non‐radiative relaxation or as fluorescence emission in a characteristic spectrum of wavelengths of lower energy. During the excitation cycle, energy is lost, that is, through molecular kinetics and vibrational relaxation.^3^ The cycle of excitation and fluorescence emission commonly occurs in the range of a few nanoseconds.^2^

In addition to non‐radiative relaxation and radiative fluorescence emission, electrons may cross over into the triplet state (*T*_1_) by intersystem crossing (ISC), a much longer‐lived excited state. From *T*_1_, an electron may return to the ground electronic state through radiative phosphorescent emission, which typically occurs on the millisecond to seconds timescale and thus is easily distinguished from fluorescence emission.^2^

In the wave description of light, the light emitted by a single fluorophore seen through an optical microscope will undergo diffraction as it travels through the microscope optics. Once the light reaches the eye or the microscope detector, it will be observed in the form of a diffraction pattern, a bidimensional representation of the response function of the instrument, also known as the *point spread function* (PSF). The PSF takes the form of a series of concentric disks (the Airy disk pattern) (Figure 1B) with the highest intensity at its center (Airy disk of order 0).^3^

The shape of the PSF depends on the wavelength of the traveling light and the numerical aperture (NA) of the microscope objective. To compare the resolving power of a microscope, the full width at half maximum (FWHM) of the PSF intensity profile is calculated (Figure 1B); the lower the FWHM value, the greater the resolving power of the microscope.^3^, ^4^

According to the Rayleigh criterion, the resolution limit at which two light‐emitting objects cannot be resolved is the distance at which the peak of the Airy pattern of one emitter overlaps with the first minimum of the Airy disk of zero‐order of the other (*R* = 0.61 (*λ_em_*)/NA)^5^ (Figure 1C). At higher NA values, the width of the zero‐order disk on the PSF equals approximately half of the wavelength of the light emitted by the fluorophore, and thus the resolution limit of an optical microscope is commonly 200–350 nm (Figure 1D)

### 1.2 Super‐resolution microscopy (SRM): A new era of optical microscopy

Fluorescence microscopy enables the observation of biological phenomena with molecular specificity. However, many aspects of these processes remain unknown due to the spatial limit of resolution of the optical microscope. The development of enhanced optical instrumentation, fluorescent probes and mathematical algorithms to overcome this limitation has accelerated considerably in recent years. These efforts have ushered in a new era for the study of nature through light: optical SRM.

The SRM era began with stimulated emission depletion (STED) fluorescence microscopy^6^, ^7^ and structured illumination microscopy (SIM).^8^ At that time, the maximum spatial resolution achieved with STED was ∼100 nm. Subsequent refinements of these approaches have further pushed the resolution limit to 20–40 nm.^9^, ^10^, ^11^ However, these techniques require highly specialised microscopes, whose cost and complexity have limited their application to a limited fraction of the bioimaging community.^1^

With the development and applications of suitable photobleaching‐resistant and photoconvertible fluorophores, single molecule localisation microscopy (SMLM) techniques were created, a set of SRM techniques based on the localisation of blinking fluorophores that obviated the requirement of a STED microscope setup^1^ (although there are some SRM variants based on STED, such as reversible saturable optical fluorescence transitions and MINFLUX [Minimal photon fluxes], which require fluorophores with drastic fluorescence fluctuation (FF) capacity such as blinking^12^, ^13^).

SRM based on single‐molecule localisation can construct a super‐resolved image because only a few emitters (around 10 emitters μm^−2^) are collected in a single frame, diminishing the probability that the fluorescence distributions of any two emitting fluorophores overlap.^14^ The position of those single emitters can be estimated with greater precision through the fitting of a Gaussian function to identify the centroid position. Since only a small fraction of the total fluorophores are emitting, it is imperative to acquire several thousands of frames of the observed field. Depending on the exact characteristics of the SMLM protocol used (such as STORM,^15^ [DNA]‐PAINT^16^, ^17^ or photoactivation localisation microscopy [PALM]^18^), the maximum resolution achievable has been reported to be in the range of up to ∼ 5 nm.^14^, ^16^

**FIGURE 2:**
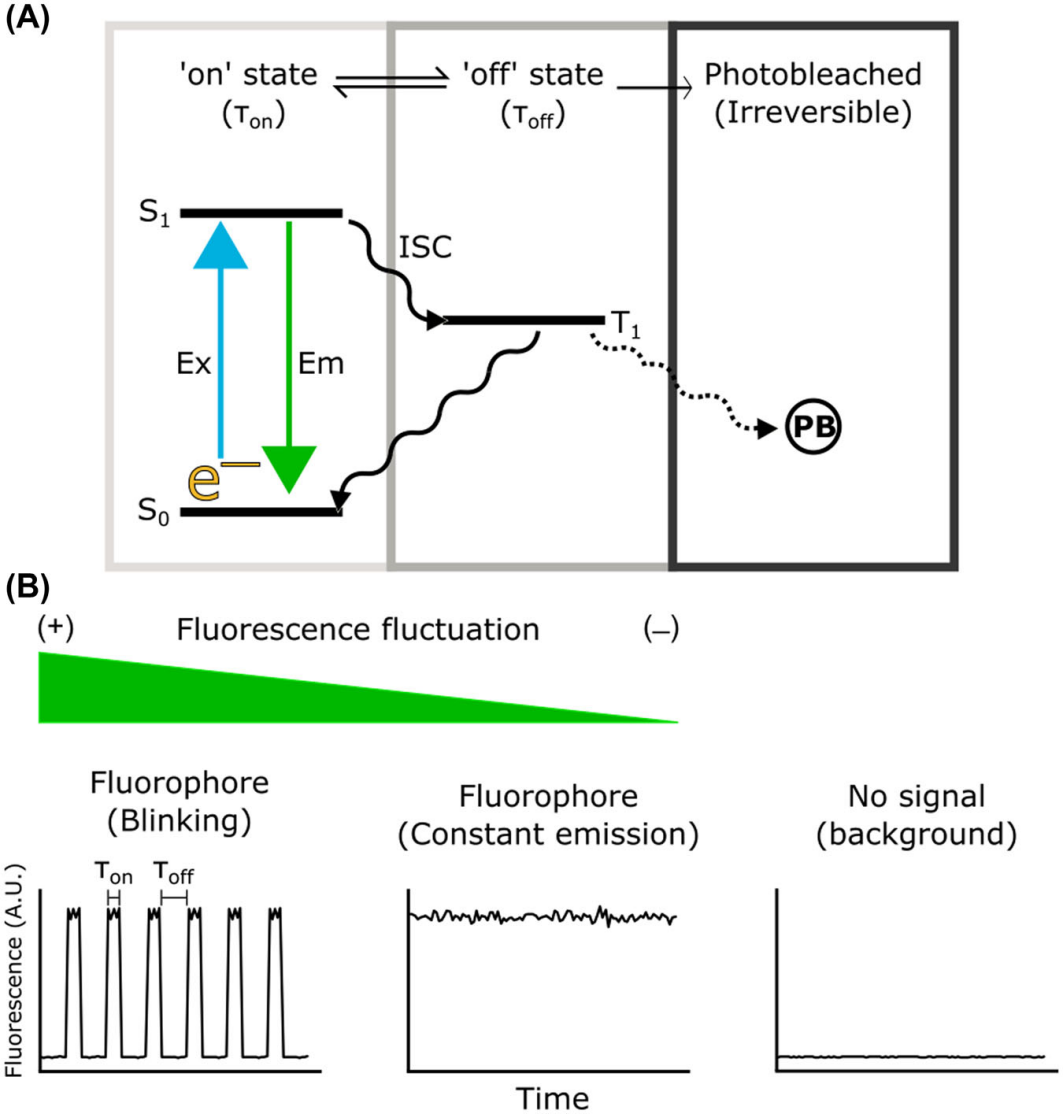
Fluorescence intermittency (A) Naturally, the fluorophores change between the ‘on’ and the ‘off" state, which manifests as a blinking pattern of emission. In some cases, a set of fluorophores transition from the triplet state to a photobleached irreversible state. Under the same fluorophore dynamics, a constant fluorescent emission is characterised by a short ‘off’ state but it also has fluorescence fluctuations (FFs) as a minor contribution. (B) FF contributions depend on fluorophore emission state. Blinking fluorophores will contribute fluctuations of higher amplitude in comparison to constant emitting fluorophores, and every detector will register variable background ‘noise’ even with no fluorophores present

SMLM requires the use of fluorophores capable of transitioning between prolonged (in the order of millisecond) emitting ‘on’ and non‐emitting ‘off’ states until eventually the fluorophore enters an irreversible photobleached state (Figure 2A). With adequate sample preparation (e.g., fluorophore selection, oxygen scavenging buffers and laser power selection) the probability of the fluorophore to transition between the ‘on’ and the ‘off’ state can be optimised to the frame acquisition rate.^14^, ^19^ Such tuning is highly experiment‐dependent, and much time must be invested to optimise the acquisition protocol for each set of experimental conditions and microscope used. Disadvantages of SMLM techniques include the need for high‐power laser illumination (from ∼62 to 7.8 kW/cm^2^),^20^ avoidance of sample drift in x, y and z positions, robust photon detection at the last set of acquired images and prevention of photodamage and phototoxicity.^1^

FF‐based SRM (FF‐SRM) relies on the statistical analysis of FFs over time.^21^, ^22^, ^23^, ^24^, ^25^, ^26^, ^27^ It can be used for blinking fluorophores in which the fluctuations are conspicuous or with more constantly emitting fluorophores where the fluctuations are generated by intrinsic variations of the fluorescence and not by prolonged ‘off’ states. In both cases, the fluorescence detected must be above the background signal^28^ (Figure 2B). The main difference between the SMLM and FF‐SRM methods is that the former's algorithms are based on the localisation of individual emitters, making SMLM more precise at the cost of more complex sample preparation and image acquisition.^29^

**FIGURE 3:**
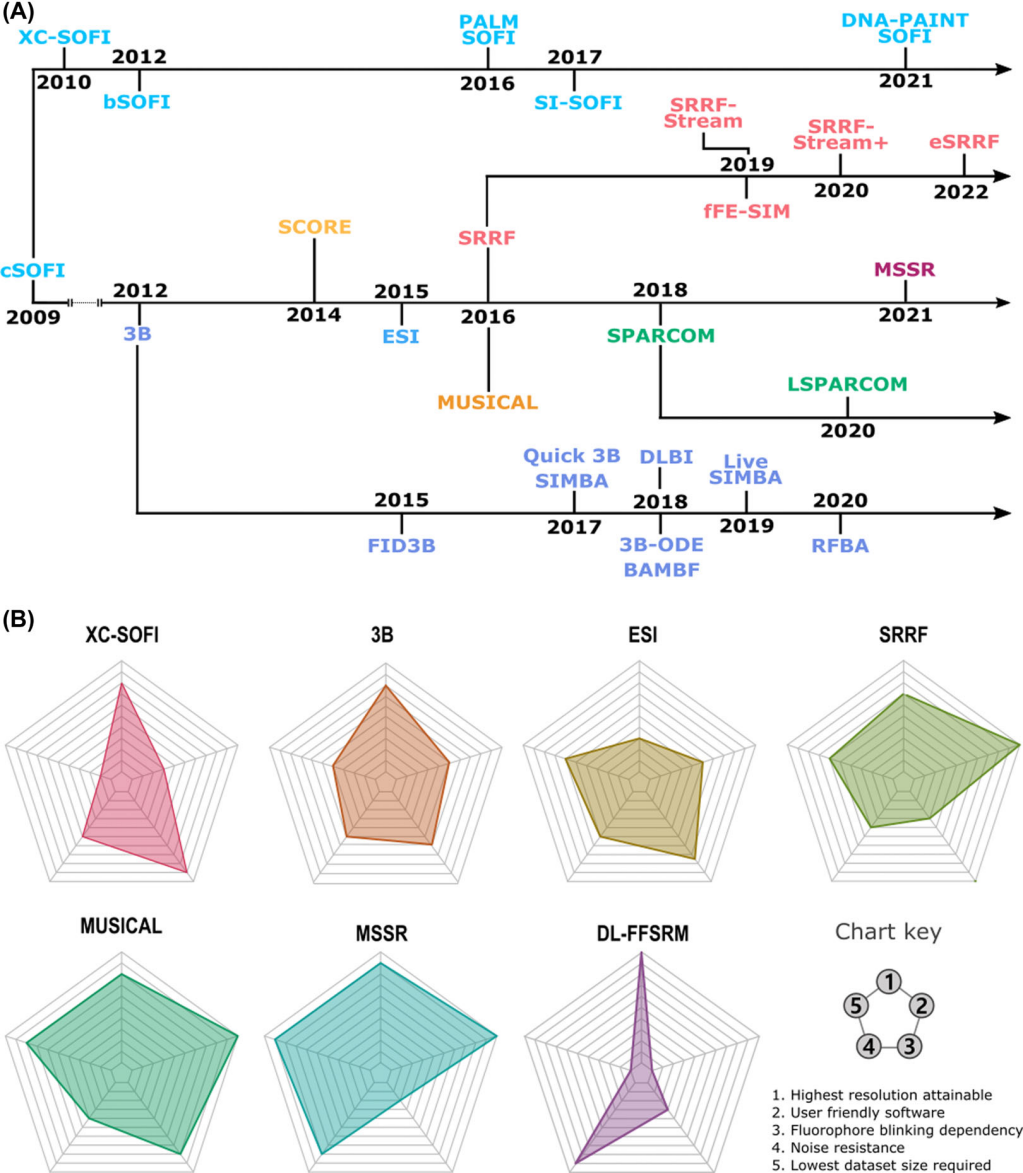
History and objective parameters related to FF based super resolution microscopy (FF‐SRM) (A) Timeline of FF‐SRM methods release. The methods located on the central branch represent their first publication and implementation. A different colour is assigned to each method and each branch from the original method contains the corresponding improved versions following the seeding colour scheme. (B) Comparative scheme of the strengths and weaknesses of the discussed FF‐SRM methods. The radar charts compare the most relevant experimental aspects to consider before FF‐SRM method selection. Higher (outermost) values are better, with the exception of spoke 3 (chart key provided in the lower‐right corner). For example, DL‐FFSRM methods offer great resistance to image noise and independence from blinking dynamics, at the cost of requiring a considerable amount of programming expertise. On the other hand, methods such as entropy‐based super‐resolution imaging (ESI) and super‐resolution radial fluctuations (SRRF) require a relatively small dataset to operate but fall relatively short in terms of the highest resolution they can achieve. Abbreviations: cSOFI, classical stochastic optical fluctuation imaging; XC‐SOFI, cross‐cumulants SOFI; bSOFI, balanced SOFI; PALM SOFI, photoactivation localisation microscopy SOFI; SI SOFI, structured‐illumination SOFI; DNA‐PAINT SOFI, DNA‐points accumulation for imaging in nanoscale topography SOFI; 3B, Bayesian analysis of blinking and bleaching; FID3B, fluorescence intensity distribution; SIMBA, single molecule guided Bayesian localisation microscopy; DLBI, deep learning guided Bayesian inference; BAMF: Bayesian multiple‐emitter fitting; RFBA, radial fluctuation Bayesian analysis; SCORE, spatial covariance reconstructive microscopy; MUSICAL, multiple signal classification algorithm; fFE‐SIM, fast fluctuation‐enhanced structured illumination microscopy; eSRRF, enhanced SRRF; SPARCOM, sparsity‐based super‐resolution correlation microscopy; LSPARCOM, learned SPARCOM; MSSR: mean‐shift super resolution; DL‐FFSRM, deep‐learning based FF‐SRM

Most of the FF‐SRM methods are compatible with epifluorescence, confocal and total internal reflection fluorescence (TIRF) microscopy as long as image sampling satisfies the Nyquist–Shannon criteria, meaning that the effective pixel size should be at least equivalent to the FWHM.^3^ As shown in Figure 3, FF‐SRM methods have been constantly improved, generating a family of FF‐SRM tools that can achieve increased spatial resolution at the nanoscopic scales.

All FF‐SRM methods work with relatively similar fundamentals; they gather nanoscopic information through an analysis of temporal fluorescence intermittency.^21^, ^22^, ^23^, ^24^, ^25^, ^26^, ^27^ Each method has particular requirements for its image acquisition strategy, which include aspects of sample preparation and imaging parameters employed such as the minimum number of frames, optical and software parameters, and image amplification, among others.^1^ Figure 3B and Table 1 summarise the strengths and weaknesses of the FF‐SRM methods described in this review.

**TABLE 1:** Features of relevant fluorescence fluctuation‐based super‐resolution microscopy methods

	Minimal frames required	Maximal resolution (nm)	Fluorophore blinking	Susceptibility to noise	Availability
XC‐SOFI	3000	50	High	Medium	Plugin for Igor Pro
3B	300	50	Medium	Medium	Plugin for ImageJ
ESI	100	∼ 120	Medium	Medium	PLUGIN for ImageJ
SRRF	100	60	Low	High	Plugin for ImageJ
MUSICAL	50	50	High	High	PLUGIN for ImageJ
MSSR	30	40	Low	Medium	Plugin for ImageJ
LSPARCOM	25 once trained	∼ 40	Low	Low	Python

Abbreviations: XC‐SOFI, cross‐cumulants SOFI; 3B, Bayesian analysis of blinking and bleaching; ESI, Entropy‐Based Super‐Resolution Imaging; SRRF, Super‐Resolution Radial Fulctuation; MUSICAL, multiple signal classification algorithm; MSSR, mean‐shift super resolution; LSPARCOM, learned sparsity‐based super‐resolution correlation microscopy.

## 2 STOCHASTIC OPTICAL FLUCTUATION IMAGING (SOFI)

Published in 2009, SOFI was the first FF‐SRM method^21^ and is considered the founder of this family. SOFI is grounded on the analysis of the temporal dynamics of FFs at nanoscopic scales. In this section, we will cover the first and second SOFI implementations.^21^, ^30^ To differentiate one work from another, we will refer to them as classic SOFI (cSOFI) and cross‐cumulants SOFI (XC‐SOFI).

**FIGURE 4:**
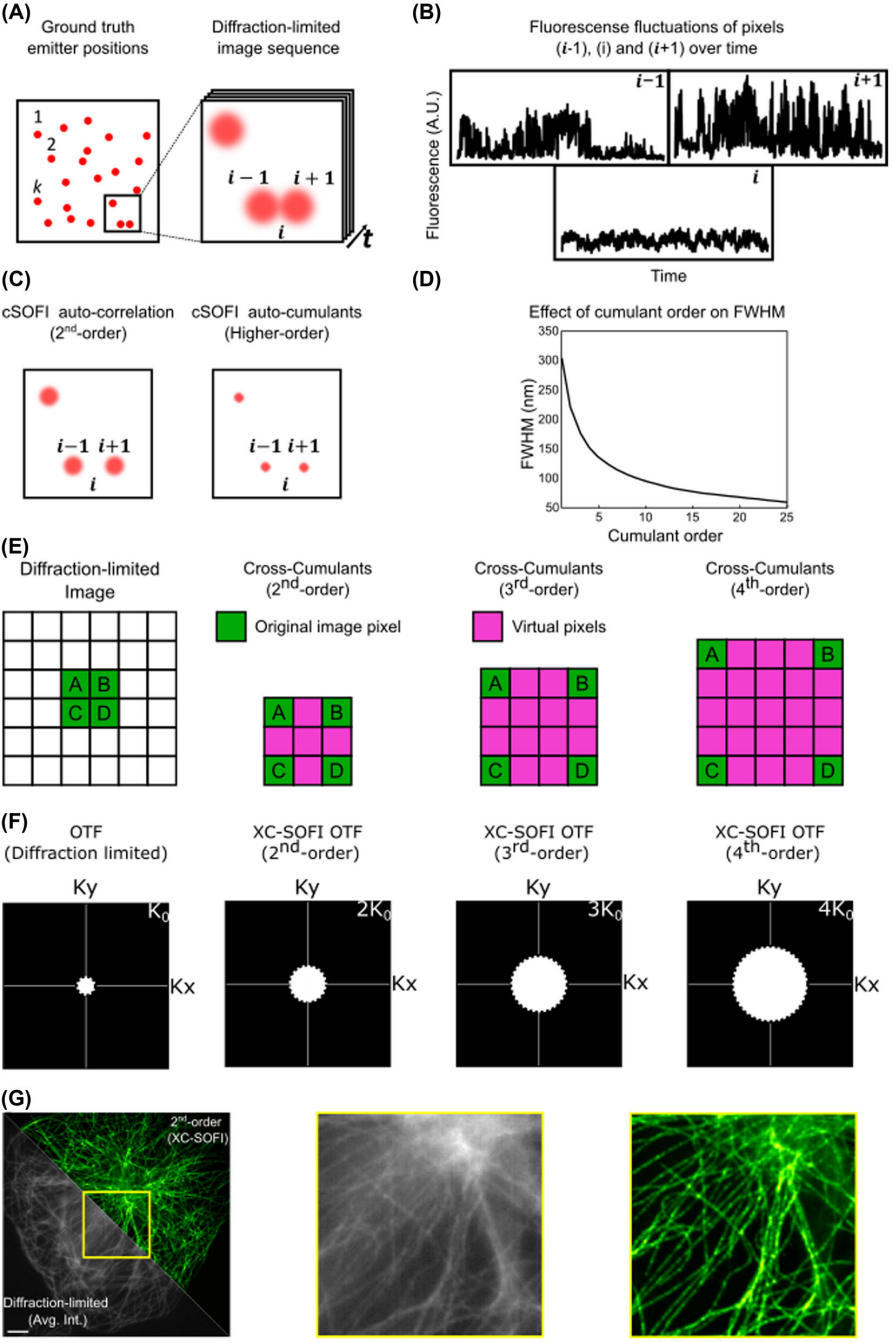
Classical stochastic optical fluctuation imaging (cSOFI) and cross‐cumulants SOFI (XC‐SOFI). (A) Original single emitter positions (ground truth) and their respective diffraction‐limited image sequence. (B) FF signal registered across time corresponding to pixels (*I* − 1), (*i*) and (*I* + 1). (C) Super‐resolved cSOFI of second and higher SOFI orders. (D) Resolution limit of cSOFI.^21^ (E) Generation of novel pixels by the cumulants in XC‐SOFI. (F) PSF in Fourier space (OTF) diffraction limited and extended by several SOFI orders. (G) Super‐resolved microtubules by second‐order XC‐SOFI. Image was reconstructed using XC‐SOFI within Localizer, Igor Pro 8.0.4.^44^ Data obtained from Sage et al.^95^ Scale bar: 5 μm

cSOFI embraces the analysis of a temporal stack of fluorescent images. It considers a fluorescence image as a digitised collection of *N* emitters, located at **r** *_k_* positions (with *k* = 1, 2, …, *N*), with constant molecular brightness. At each pixel, the fluorescence signal fluctuates in a time‐dependent manner, due to the fluorescence intermittency of the emitters it harbours. Within a single fluorescence image, each emitter is considered to be convolved with the PSF of the optical system. Hence, a cSOFI experiment encompasses the analysis of a temporal sequence of images gathered from a static scene, in which the FF contains nanoscopic information not available in the spatial domain of a single diffraction‐limited image (Figure 4A).

An ideal sample for a cSOFI experiment is one for which the fluorophores are spatio‐temporally static (i.e., no sample drift) and FFs due to transition through excited states are the main cause of change in the fluorescence signal. The fluctuations from the background (pixel *i*) are different from the FFs; hence, they can be separated (Figure 4B). To separate the fluorescence from the background, cSOFI seeks temporal self‐similarities of the signal of each pixel with itself at a time *τ*, which is achieved through computing the temporal autocorrelation function or related mathematical treatments.^21^ This is the elemental form of cSOFI, which improves the resolution by a factor of $\sqrt{2}$ and it is called second‐order SOFI (Figure 4C).

For further resolution enhancement, it is possible to compute cSOFI of higher orders (*n* ≥ 3). This approach does not auto‐correlate the FF signal; instead, it computes further statistical descriptors of the fluorescence dynamics called temporal cumulants, which are similar to the statistical moments.^31^ Higher‐order auto‐cumulant cSOFI improves the resolution by a factor of $\sqrt{\;n}$, where *n* is the order of cSOFI (Figure 4C). In theory, a fourth‐order cSOFI generates a two‐fold improvement in resolution ($\sqrt{\;4}=\;2$), and a 16th‐order cSOFI can achieve a four‐fold resolution increase ($\sqrt{\;16}=\;4$). Irrespective of the order of cSOFI used, it has been demonstrated that the maximal experimental cSOFI resolution is ∼ 60 nm (Figure 4D).^21^

The increase in resolution in cSOFI can be scored by measuring the reduction of the PSF as a function of the order of cSOFI. However, a limitation of cSOFI is that the image pixel size is constant between the input diffraction‐limited dataset and the super‐resolved image. This issue imposes a boundary to the achievable resolution.^30^ If the pixel size of the diffraction‐limited dataset is oversampled (greater than two pixels covering the full width of the zeroest‐order peak of the PSF), higher orders of cSOFI will deliver images with enhanced spatial resolution. However, if the pixel size of the diffraction‐limited dataset is exactly at the limit specified by the Nyquist–Shannon sampling criterion or undersamples it (fewer than 2 pixels covering the zeroest‐order peak of the PSF), the gain of resolution provided by higher orders of cSOFI will be nullified due to the undersampling of higher spatial frequencies.

To generate a super‐resolved image with a greater number of smaller pixels, XC‐SOFI replaces the auto‐cumulants approach and instead applies a cross‐cumulants analysis.^30^ This mathematical modelling approach generates novel virtual pixels that increase in number and reduce in dimension with the order of XC‐SOFI (Figure 4E). The amplification of the original image generates a checkerboard effect. To circumvent this problem, the fluorescence intensity assigned to each virtual and original pixel is modulated by a distance factor. At this point, a preliminary image of XC‐SOFI has already been generated. The XC‐SOFI algorithm finalises with a deconvolution step that balances the contrast of the images, facilitating the observation of the gained spatial frequencies.

In the Fourier space, the objective lens of a microscope acts at a finite aperture that limits the spatial frequencies of the diffraction‐limited image. This can be represented by the optical transfer function (OTF), which is itself the Fourier transform of the PSF. The Abbe criterion and the NA of the lens can be directed linked by the maximum observable spatial frequency *K*_0_ = 2 NA/*λ_em_*, which can be conveniently represented by a circle of radius *K*_0_ within the real part of the OTF (also called the modulation transfer function; Figure 4F, white dashed circles) and reviewed by Vangindertael and colleagues.^3^, ^29^, ^30^, ^32^

The deconvolution step used by XC‐SOFI is a Wiener deconvolution.^30^, ^33^ Figure 4 shows the maximal spatial frequency obtained after each order of XC‐SOFI followed by Wiener deconvolution. This strategy extends the *K*_0_ radius by the SOFI order (K_0_ •n). The resolution enhancement of XC‐SOFI is *n* rather than $\sqrt{n}$ as in cSOFI.^29^, ^30^, ^32^

cSOFI and XC‐SOFI have shown capabilities for 3D SRM by analyzing Z‐stack datasets acquired on a widefield microscope.^34^ Moreover, XC‐SOFI has also been used for dual‐colour SRM.^35^ SOFI has been used in several biological models and the theory has been developed far beyond the seminal publication of cSOFI and XC‐SOFI. In 2012, the mathematical principle of XC‐SOFI was restructured with special consideration of molecular brightness, on‐time ratio, and density of fluorophores; as a result, balanced SOFI (bSOFI) was published.^36^

In 2014, bSOFI, was applied to images acquired by a custom microscope capable of multi‐channel and simultaneous multi‐plane imaging of fixed 3D mitochondria networks in C2C12 cells and live‐cell imaging of HeLa cells.^37^ A combination of PALM^18^ with bSOFI^36^ was reported for the imaging of focal adhesion dynamics, and by combining both methods on the same data, the authors generated an improved SR image in comparison with each super‐resolution method applied independently.^38^

Meanwhile, in another merger of techniques at the fluorophore level, the combination of bimolecular fluorescence complementation^39^ with the photoswitchable fluorescent protein Dronpa resulted in a novel strategy for determining protein–protein interactions in live cells, with the application of XC‐SOFI analysis to obtain the nanoscopic position of the interacting proteins.^40^

SOFI does not demand the use of controllably blinking fluorophores due to the analysis of FF over time. Nonetheless, with better blinking kinetics, the more significant improvement in resolution in the super‐resolved image.^28^, ^29^

The number of frames required to reconstruct a super‐resolved image using SOFI depends on the SOFI variant, and on the SNR and the desired SOFI order to be calculated, that is, 3000 to 5000 frames for the third order and >10,000 frames for the fourth order. Like other SRM methods, SOFI is not artefact‐free and one should be cautious when interpreting the super‐resolved image. The math behind second‐order SOFI relies only on positive values, while the third‐ or higher‐order SOFI involves negative values that are prone to cause a type of artefact called ‘cusp’.^41^ It has been proposed to use statistical moments and local dynamic range compression to mitigate cusp artefacts.^42^

Several software packages are available for a SOFI analysis. Localizer is an open‐source software optimised with the most recent version of XC‐SOFI^43^; it is accessible as a MATLAB script or a user‐friendly plug‐in for Igor pro.^44^ bSOFI can be executed as a MATLAB script^36^; and recently, open‐source Python implementations of SOFI have been published (PySOFI).^45^ For microscopists interested in delving deeper into the principles and applications of SOFI, we suggest reading a recent and exhaustive SOFI review.^32^

## 3 BAYESIAN ANALYSIS OF BLINKING AND BLEACHING (3B)

**FIGURE 5:**
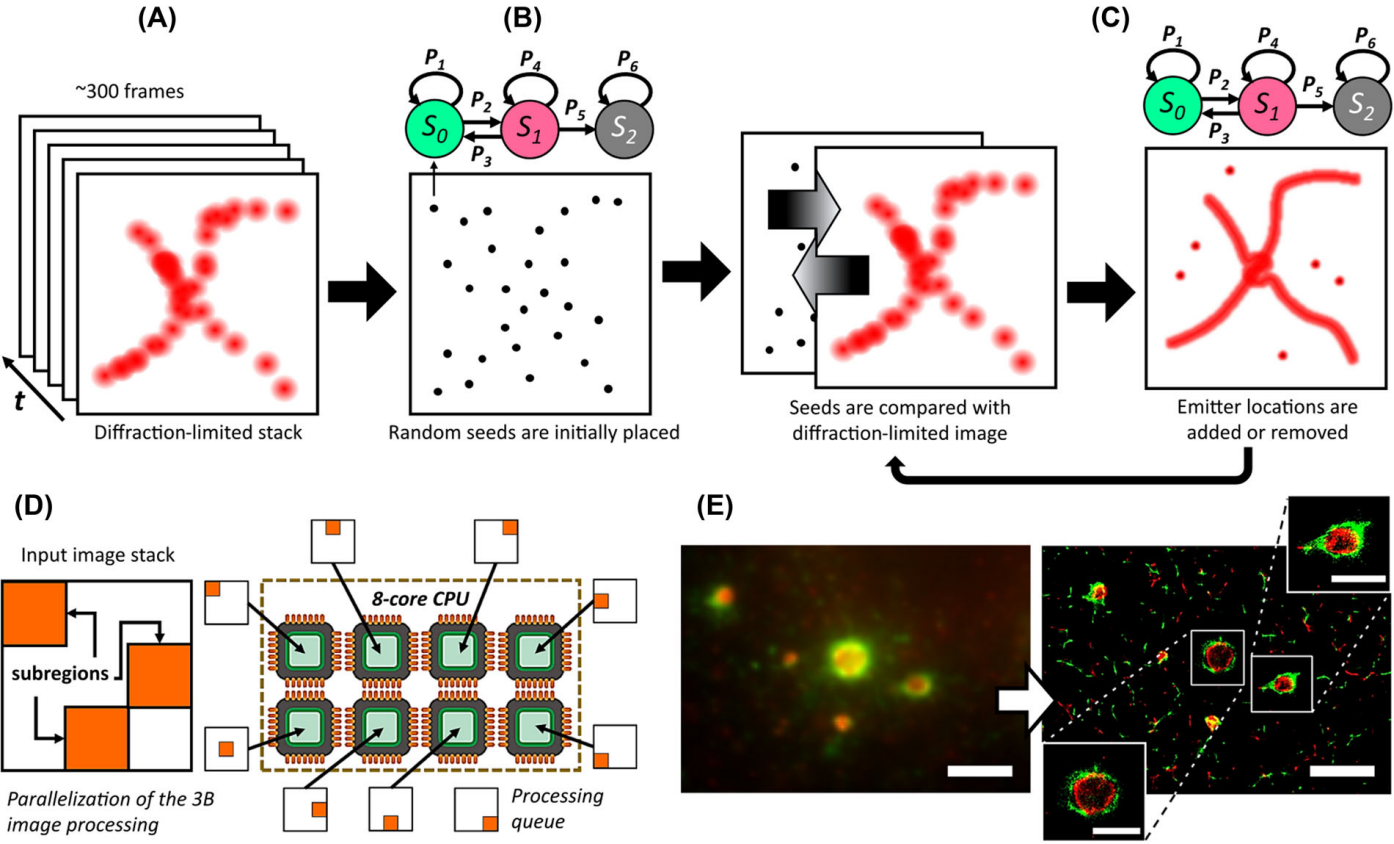
Bayesian analysis of blinking and bleaching (3B) microscopy. (A) The input data are a diffraction‐limited stack of fluorescence images containing ensemble fluorophores undergoing a time‐dependent blinking due to transitions towards excited (*S*_0_, *S*_1_) and dark states (*S*_1_, *S*_2_). (B) First, the initial model of the emitters’ positions is created by placing random seeds over the analysed region, where each seed represents a fluorophore. (C) For each seed placed in the model, the behaviour over time is sampled based on the transition matrix and the initial probability as a Markov chain Monte Carlo sampling. Local decisions are made by adding and removing one fluorophore at a time from the model. Using Bayesian Inference, the probability that the model with M fluorophores (*F_M_*) generates the diffraction‐limited data is computed. Finally, the SR image is created by accumulating the quantised positions of the probability map to the nearest pixel in the high resolution density image, applying a blurring to each fluorophore. (D) The parallelised version of 3B allows for multi‐core parallel processing of subregions (or ‘patches’) of the input image stack, which aims for reduced computation times. (E) Super‐resolved micrography using the parallelised version of 3B^49^, ^52^ of rotavirus viroplasm where the green and red channels correspond to the VP6 and NSP2 proteins, respectively. Scale bars: 2 μm, insets: 1 μm

3B estimates the position of the molecules using Bayesian inference, factorial hidden Markov chains, and Markov chain Monte Carlo (MCMC) sampling (Figure 5A).^22^ 3B works with a high density of fluorophores, that is, each frame can contain overlapping fluorophores, and may require as few as 300 images to compute an SRM image.^22^, ^46^ For this reason, 3B can be used for both fixed or live‐cell imaging on wide‐field images of samples expressing typical levels of fluorescent proteins, achieving a spatial resolution of ∼ 50 nm.

In the 3B analysis, each fluorophore is modelled as a Markov chain transiting between three possible states: *S*_0_, *S*_1_ and *S*_2_ (Figure 5B). *S*_0_ and *S*_1_ are excited states. Emission of fluorescence takes place at the *S*_0_ state, whereas the *S*_1_ state encompasses transition towards non‐fluorescent excited states such as the triplet state. The *S*_2_ state indicates that the fluorophore has reached an irreversible dark state (photobleached). The transition between states is characterised by transition probabilities (*P_n_* where *n* = 1, …, 6 as depicted in Figure 5B). Throughout the image acquisition, the fluorophores in *S*_0_ or *S*_1_ can persist in the same state (*P*_1_ and *P*_4_, respectively), or switch between *S*_0_ and *S*_1_ (*P*_2_ and *P*_3_). Fluorophores that have entered the *S*_2_ (*P*_5_), cannot regress to *S*_0_ or *S*_1_ (*P*_6_). These transitions define the transition probability matrix that is related to the temporal stochasticity of fluorescence dynamics.

The 3B algorithm initialises by placing random seeds, assuming that fluorophores have the same probability of appearance over the entire region of analysis (Figure 5B). In the following step, the model is computed for each added fluorophore, F, and it is compared with the given input data (stack of images), D. These seeds are removed or added in such a way that the proposed model approaches the input data (Figure 5C). A set of models are generated with a different number of fluorophores to maximise the probability of the model, compared to the input data.

In summary, the comparison is performed by adding or removing fluorophores in the entire region, making local decisions, and then the model is re‐optimised until the algorithm is stopped. Model convergence is said to be achieved when there is no significant variance between reconstructions of consecutive iterations (200 iterations are recommended for model convergence).^22^ The reconstructed super‐resolution image is a probability map of the position of the fluorophores, where the intensity of the pixels depends on the probability that a fluorophore is located in the region of interest.

When the 3B analysis was released, the execution required a sufficient level of programming skills; nevertheless, an Image‐J plug‐in was developed.^22^, ^47^ Both the script and the plug‐in have the drawback of the high computation time required to analyse a set of images. The Image‐J plug‐in still required 6 h to analyse a 1.5 × 1.5 μm area with 200 frames in the stack.^22^, ^47^ Despite the extended compute time, the Image‐J plug‐in is user friendly since it only requires information about the FWHM associated with the PSF, the pixel size (in nm) and the initial number of seeds (region of Interest [ROI] width per height/10), which is an estimation of the number of emitters in the ROI.

Subsequent developments of 3B aimed to reduce the computational cost by parallelising the analysis with methods such as cloud computing^48^ and cluster computing or in a conventional personal computer (Figure 5D).^49^ Since the computation time for 3B depends on the size of the images to be analysed, one way to reduce the computational cost was to divide the size of the images to be analysed by regions of smaller size; in this way, each region is analysed separately. In the end, the SR (super‐resolution) image is generated by stitching the results of each SR region. As this process can result in artefacts in the SR image, a study of the optimal overlap between the ROIs was carried out to reduce these artefacts.^49^

A more recent development, Bayesian localisation microscopy based on fluorescence intensity distribution performs a statistical analysis to improve the accuracy of the initial seeds of the emitters based on the pixel intensity, reducing the computational time and improving the SR image.^46^ Later, Quick‐3B was developed. It combines the *k*‐means clustering algorithm and a modified 3B analysis with a limited forward algorithm, which accelerates computation time 17‐fold, compared to 3B.^50^ Also in this work, single molecule‐guided Bayesian localisation microscopy (SIMBA) is presented as a way to reduce the discontinuous structures that the 3B method creates as artefacts.^50^ However, SIMBA requires two types of emission fluorescent signals, using an SMLM algorithm (PALM) as an initial guide for the Bayesian analysis. Recently, Live‐SIMBA was published, a plug‐in for ImageJ based on SIMBA that does not necessarily require a dual‐channel dataset. It is characterised by a computation time reduced thousands of times, compared with 3B, and an acceleration 25‐fold, compared to SIMBA.^51^

In 2019, another variant of the original 3B algorithm (3B‐ODE: 3B‐Ordinary Differential Equations) was presented to improve the convergence of the model, and the accuracy for estimating the probability map of the fluorophore positions.^52^ It consists in calculating the transition probabilities between states by fitting the experimental data with ordinary differential equations. 3B‐ODE models and fits velocity constants for the electronic transition between on and off state experienced by the fluorophores within the diffraction‐limited image stack.^52^ The Bayesian multiple‐emitter fitting (BAMF) is also based on a 3B algorithm, which uses reversible jump MCMC combined with MCMC sampling. The advantage of BAMF is that it incorporates the photophysical information of the sample and the density of the emitters for the creation of the model as prior information, allowing the adjustment of multiple emitters and removing the heterogeneous background. This method also provides the uncertainties in the number of emitters and the locations of the most likely model.^53^

Finally, radial fluctuation Bayesian analysis is a proposed method that uses light‐sheet microscopy with Bessel plane illumination for 3D SR imaging.^54^ For this, the initial points of the 3B algorithm are calculated using super‐resolution radial fluctuations (SRRFs, see Section 5) and the model optimisation is based on these same locations generated by SRRF, reducing the computation time to half of that required for 3B.

3B analysis is a powerful technique that can be used for live‐cell imaging of samples labelled with standard fluorescent proteins. In a wide‐field experiment, relatively few frames (hundreds) are needed to reconstruct a super‐resolution image. It can accommodate overlapping of fluorophores and achieves a resolution of ∼ 40 nm.^52^ All current 3B analysis implementations require programming skills (except the ImageJ plug‐in, canonical 3B and Parallel3B) and, in some cases, are no longer being maintained or there is no open‐source implementation available. Overall, the main drawback of the 3B analysis is the high computation time. However, this problem has been addressed by improvements or optimisations in the algorithm or by adjusting the parameters such as the number of initial seeds or the transition matrix for a faster model convergence.

## 4 ENTROPY‐BASED SUPER‐RESOLUTION IMAGING (ESI)

At the time of the publication of ESI,^23^ improved versions of cSOFI and 3B had been published that solved some problems from their original publications, like the consideration of non‐linear brightness, blinking of the fluorophores and the computational time to analyse the input data.^36^, ^48^

**FIGURE 6:**
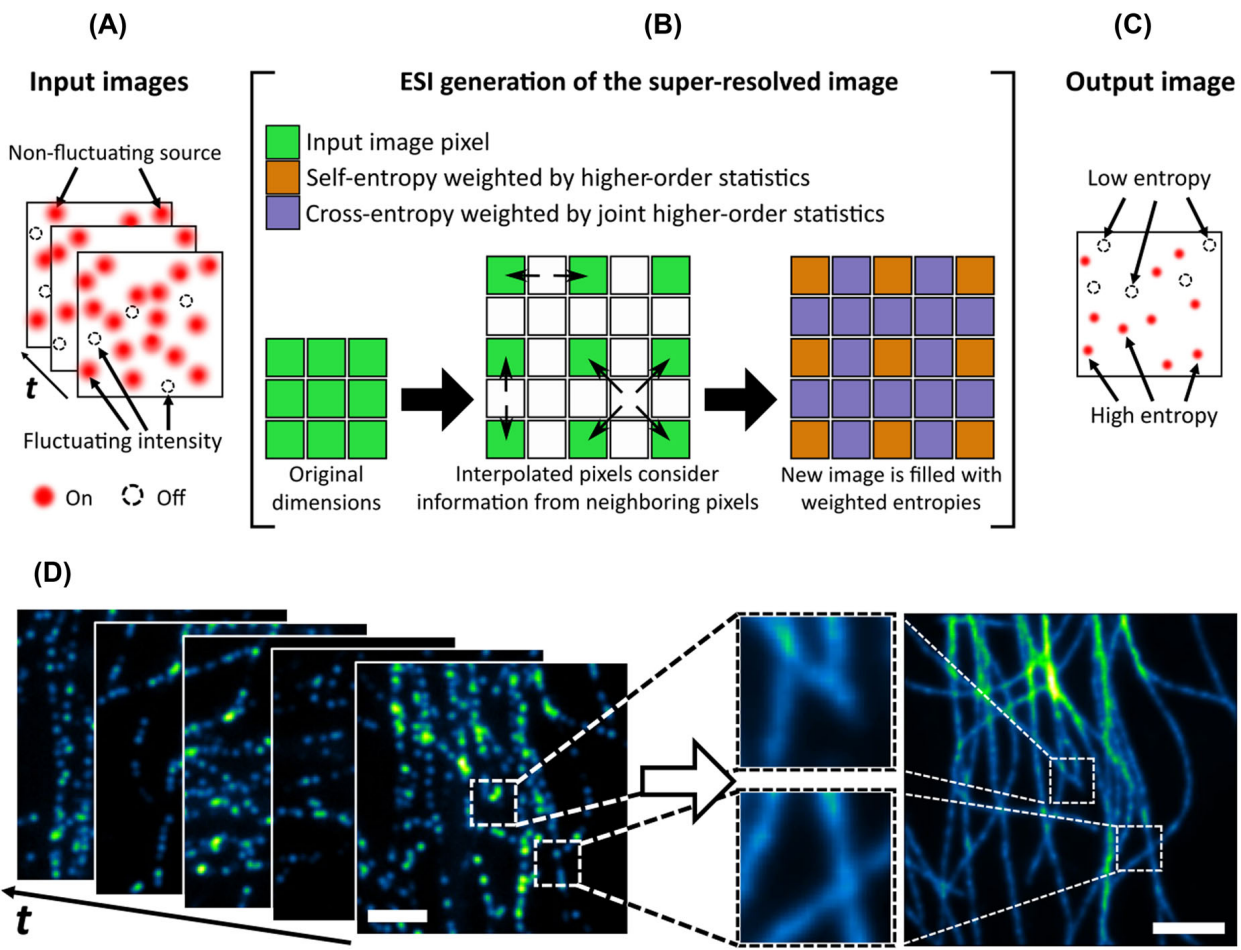
ESI. (A) The input image stack consists of a set of fluctuating and non‐fluctuating fluorophores. (B) The super‐resolved ESI image is created by interpolation of the purple pixels in between the corresponding original green pixels. The orange pixels in the new image have a value that corresponds to the information content of the original image pixels (green), while the interpolating pixels (purple) consider the information of their neighbors. This scheme represents a single iteration; more iterations can be performed by using several output ESI images as new input. (C) The resulting image contains only the information of those emitters whose degree of variation (entropy) was high enough for detection. (D) Super‐resolved micrography of simulated microtubules.^95^ Example generated using the ESI plugin for ImageJ with parameters: output images = 100, bins for entropy = 100, order = 1, multicore = enabled. Scale bars: 2 μm

ESI was the first FF‐SRM method that required only 100 frames collected in a fluorescent scene to reconstruct a super‐resolution image.^23^ This is achieved by analysing FFs through the local information content, pixel‐wise, of the temporal dynamics of the fluorescences stored in the image stack. The rationale of ESI is that, in a time‐lapse stack, each pixel harbours the intensity fluctuation of a specific place in space. The information‐based entropy depends on the frequency of occurrence of fluorescence events, and by doing so, a highly fluctuating source will have high entropy, while a constant signal (such as a non‐blinking emitter) or a background pixel would be discarded (Figure 6A). If a fluorophore is found in a pixel, its temporal fluctuations will contain more information (entropy) than the background due to the photon emission process.

ESI, like SOFI and its variants, uses a pixel‐wise calculation to extract the temporal information, with the difference that it calculates the information‐based entropy,^23^ instead of cumulants as in SOFI.^21^ While cumulants are an alternative to moments of a distribution (fluorescence intensities in this case), entropy measures the information content of the fluorescence signal. This entropy is weighed by the central moment (moments of the mean) of order *n* in the pixel, meaning that the central moment of order 2 is the pixel variance. Also, it is important to note that the second and third central moments are equal to the second and third cumulants, respectively, although at higher orders moments and cumulants generally differ.

A similarity between the ESI and SOFI is that ESI generates virtual pixels by exploiting the cross‐entropy of neighbouring pixels (combined with higher‐order statistics),^23^ and XC‐SOFI generates extra pixels by computing the cross‐cumulants of neighbouring pixels.^30^ Since the cross‐entropy is non‐commutative, ESI calculates the average of the two possible cross entropies between two pixels. In the process of generating a new super‐resolved image, ESI creates a magnified grid of the original image by interpolating one pixel in between each original pixel as shown in Figure 6B.

Regarding their ability to reduce the PSF width (meaning enhancing resolution), while SOFI directly raises the PSF to the order *n* of the cumulant (thus achieving improvement of √*n* in resolution for cSOFI and of *n* for bSOFI),^21^, ^36^ ESI considers 2*n* while obtaining the higher‐order statistic of the pixels, and the authors show that ESI improves the resolution by √(2*n*).

In ESI, the pixels in the new grid that correspond to the original image (green) are assigned the value of their entropy weighted by a higher‐order statistical measure selected by the user. This higher‐order statistic is the central moment of the mean, meaning that an order of *n* = 2 will yield the variance at that specific pixel. For the interpolated pixels, their new value is calculated from the cross‐entropy of the neighbouring pixels weighted by the joint centralised moment of those same neighbouring pixels. Following the example of XC‐SOFI, this cross‐correlation between neighbouring pixels yields a true signal in contrast to a typical interpolation on the final image (Figure 6C,D)

ESI has been deployed as a plug‐in for ImageJ, which generates a 2X increase in spatial sampling per iteration of the analysis.^23^ In this implementation, each new iteration takes the ESI stack (output) and uses it as input, so ESI will calculate the entropy and cross‐entropy of this new input data. No more than three iterations are recommended due to the non‐linear decrease in contrast, as well as information loss by pixel size reduction.^23^ The plug‐in also requires the number of images in the output that will define how the initial stack will be subdivided to generate the SR images.

Overall, ESI is an FF‐SRM approach that can work with as few as 100 wide‐field images to provide reconstruction with narrower structures. The combination of orders and iterations remains user‐dependent, which can be adjusted for achieving noise suppression; however, there is always the possibility that this suppression eliminates signals from fluorophores. In addition, the iterative process itself has a drawback since the plug‐in is restricted to produce only a two‐fold increase in spatial sampling and analyses only a fraction of the frames per iteration when more than one image in the output is desired. Additional settings to aid the reconstruction had included the use of chip‐based waveguides for alternative illumination set up.^55^

## 5 SUPER‐RESOLUTION RADIAL FLUCTUATION (SRRF)

**FIGURE 7:**
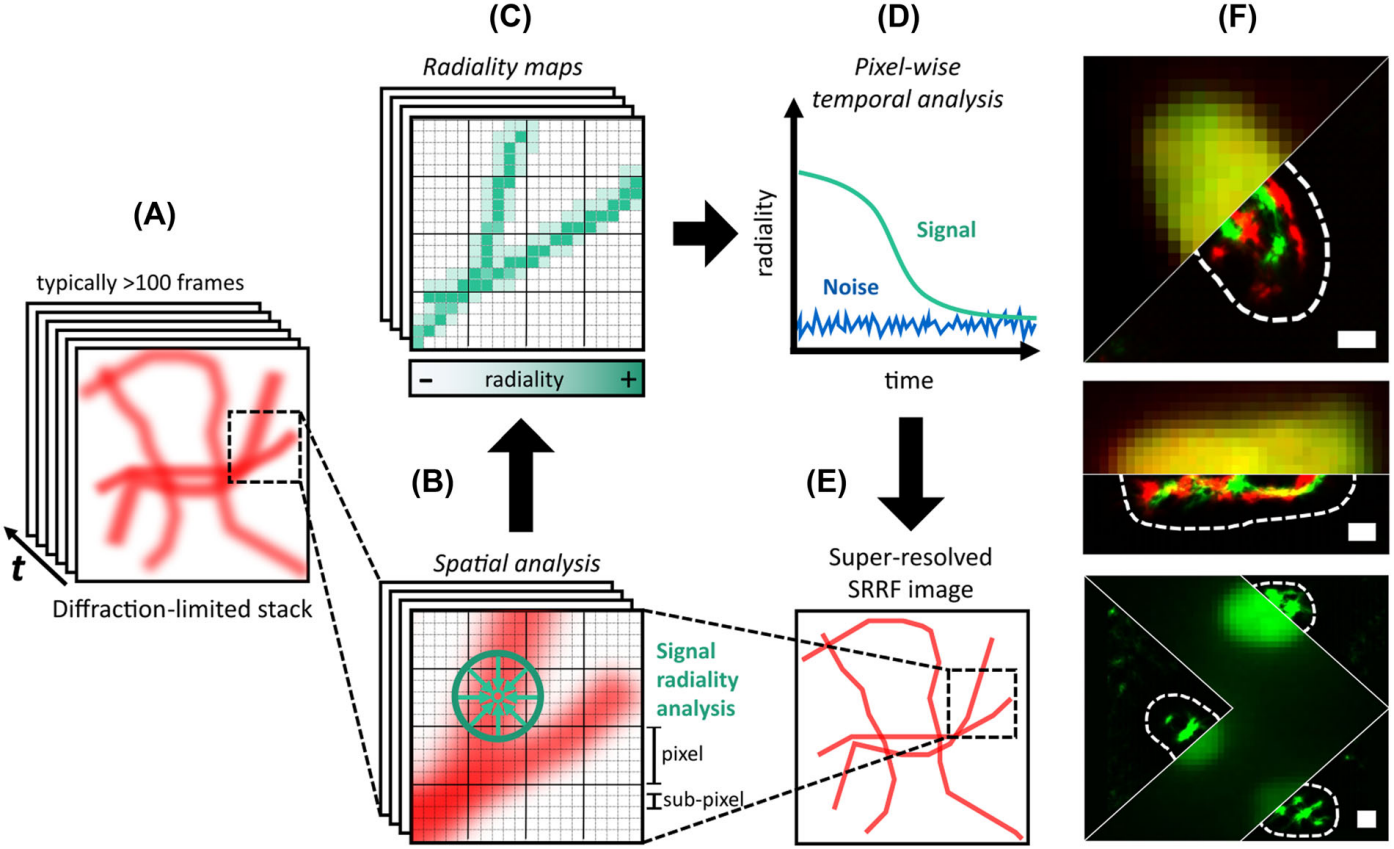
Super‐resolution radial fluctuations. (A) Each image of the input sequence is (B) subpixel interpolated and on each of them a radiality measure is performed. (C) A radiality map is generated on every image from the sequence and (D) they are correlated along time in order to (e) form the reconstructed image. (F) Super‐resolved images of *Escherichia coli* imaged on HILO microscopy, where the green and red channels correspond to mClover3 and mRuby3, respectively. Reconstructions were generated with the NanoJ SRRF plugin of ImageJ /FIJI with the parameters: Ring radius = 0.5, magnification = 10, axes in ring = 8. Scale bars: 350 nm

SRRF was developed in 2016,^24^ and consists of two main parts: a spatial analysis in which the algorithm generates a radiality map per each raw image and a temporal analysis of each radiality map by higher‐order temporal statistics to generate a single super‐resolved image (Figure 7A–C).^24^ SRRF is capable of reconstructing an SRM image with only 100 frames, achieving lateral resolution of ∼ 60 nm on TIRF, confocal laser scanning microscopy, wide‐field and traction force microscopy datasets.^24^, ^56^, ^57^

The spatial analysis of SRRF begins with the generation of digital subpixels per each ‘real’ pixel of the raw image sequence by a bicubic interpolation (Figure 7B). Next, each subpixel is assigned a value according to the probability that it has an emitting fluorophore. This value is generated by radiality maps (Figure 7C), which measure the degree of convergence of intensity gradient vectors on one subpixel basis. If the subpixel has a fluorophore, the convergence of the vectors will be higher than those coming from the image background.

The radiality maps can achieve better results for the SRM reconstruction by using a smaller ring radius and more axes in the ring. On the SRRF ImageJ plug‐in (NanoJ‐SRRF), the default ring radius is 0.5 pixels and the number of axes is six. It is important to consider that modification in these values will affect the computing time, the resolution achieved and the propensity to generate artefacts on the final SRM reconstruction.^24^, ^56^ The maximal digital increase generated by SRRF (defined by the authors as ‘magnification factor’) is 10x, and users can define it according to their necessities.

Within a single radiality map, SRRF is capable of distinguishing two fluorophores separate by 0.7 the FWHM of the PSF; however, the noise of the image can be interpreted as a fluorophore signal and generate artefacts. Temporal analysis is needed to mitigate artefacts as the radiality peak on the background subpixels will be uncorrelated over time (Figure 7D). Like the other FF‐SRM methods, the temporal analysis is affected by the sample movement, thus the NanoJ‐SRRF plugin allows the application of a drift correction table, defined by the user or calculated by the algorithm.

SRRF encompasses any of four temporal analyses: temporal radiality maximum (TRM), temporal radiality average (TRA), temporal radiality pairwise product mean (TRPPM) and temporal radiality auto‐cumulant (TRAC).^24^ The temporal analysis method should be chosen depending on the acquired data; for example, TRA is recommended with noisy images, and TRM is preferred when the images contain constantly emitting sources. Either TRA or TRM denoise the radiality map because they project the maximum and average values from the stack of the radiality maps. The TRPPM and TRAC orders 2, 3 and 4 (similar to those used in cSOFI) are higher‐order statistics methods that improve contrast, fidelity and resolution on the SRM image.^24^

SRRF has been extensively utilised in bioimaging because it does not require the use of specific fluorophores, buffers or microscopes, and it only needs 100 frames to reconstruct a super‐resolved image. It is compatible with live‐cell imaging since the use of high illumination power is unnecessary, which diminishes phototoxicity on the sample. SRRF has been used in combination with SIM (fFE‐SIM),^58^ stochastic optical reconstruction microscopy (STORM),^59^ AiryScan (FEAST) and expansion microscopy with Airyscan^60^ achieving resolutions of ∼ 32, ∼40 and ∼26 nm, respectively.

Unfortunately, SRRF is prone to generate artefacts on noisy images and very high‐density fluorophore samples, and often over‐narrows the structures on the image.^32^, ^61^ Therefore, it is recommended to optimise the input parameters (especially ring radius, axes and temporal analysis) with error mapping approaches.^62^

An improved version has been released named enhanced SRRF (eSRRF),^24^, ^63^ which improves the original algorithm by changing three main aspects; the first corresponds to the interpolation performed to generate subpixels in eSRRF—this process is performed by Fourier transform interpolation, which minimises the artefacts in the final SR image. Second, the radiality maps are now calculated by radial gradient convergence (RGC), which is calculated by a weighted factor map based on the radius (*R*‐value) defined by the user and the intensity gradient of each pixel on the original image. Finally, a new parameter defined as sensitivity (*S*‐value) is included that allows better control of the PSF sharpening performed by the RGC.

The eSRRF plug‐in has become more user‐friendly by adding a parameter ‘sweep’ by which different values of the radius and sensitivity are compared using the error and mapping tool SQUIRREL, diminishing the probability of generating artefacts by a non‐optimised selection of the algorithmic parameters. In general, low sensitivity and radius values increase image fidelity; in contrast, the resolution is increased with higher *S*‐values at the cost of lower image fidelity.

Currently, SRRF and eSRRF are available as ImageJ /FIJI plug‐in NanoJ SRRF and NanoJ eSRRF.^24^, ^63^ In addition, SRRF has been deployed in python,^59^ with a decrease of up to 78‐fold for the processing time (compared with the imageJ SRRF plug‐in) by allowing parallel computing supported by compute unified device architecture (CUDA; code not available). SRRF can be performed in real‐time with parallel GPU (Graphics Processing Unit) computing with SRRF‐Stream and SRRF‐Stream+, which are only available for microscopes with specific Andor and Sona cameras.

## 6 MULTIPLE SIGNAL CLASSIFICATION (MUSIC) ALGORITHM (MUSICAL)

**FIGURE 8:**
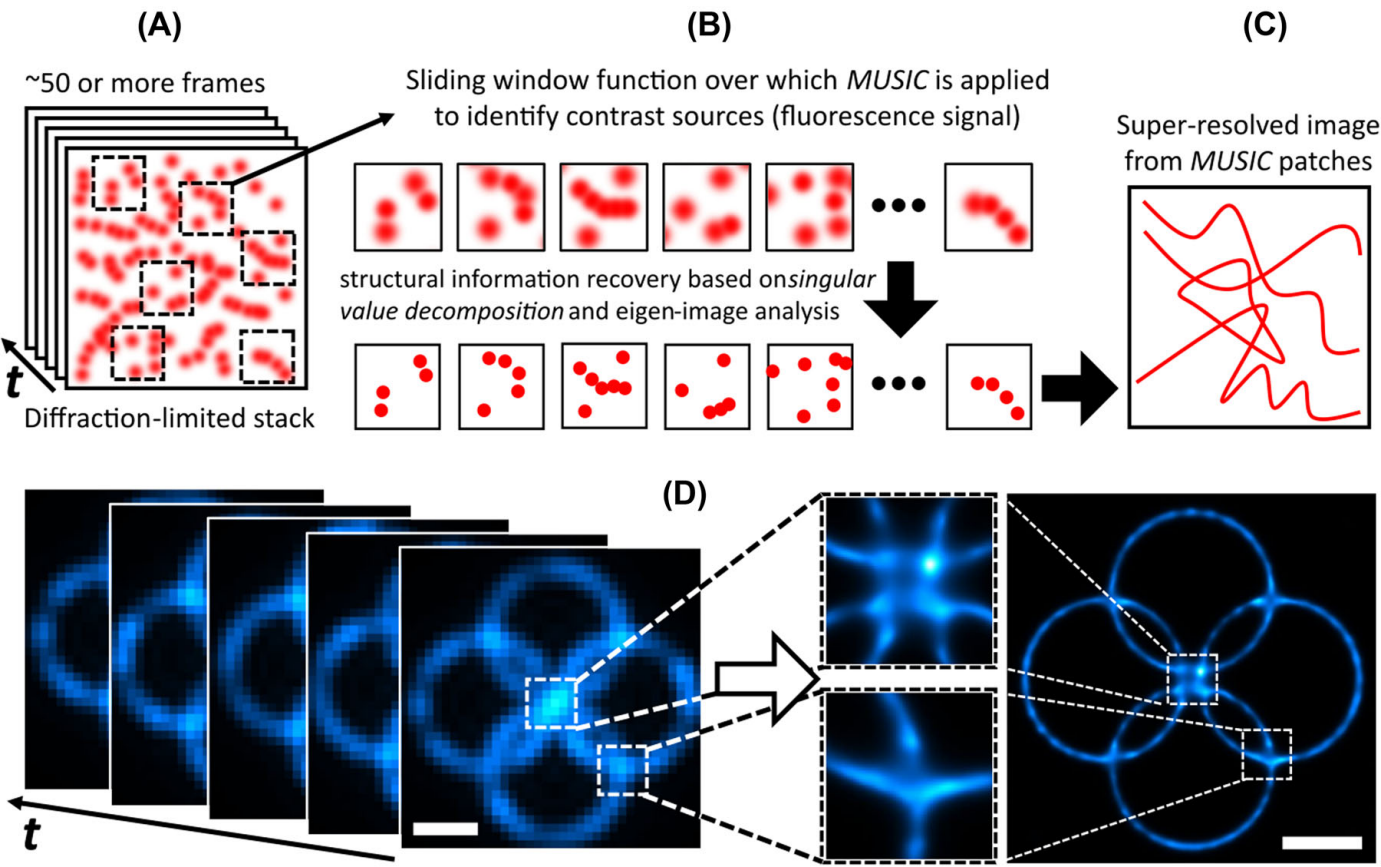
Multiple signal classification algorithm. (A) For every pixel of the input image stack, a spatial window with a size that is approximate to the full width at half maximum the microscope's PSF (in pixels) is considered. This window is then scanned across the whole image. (B) This generates multiple temporal image ‘patches’, over each of which singular value decomposition is performed and the eigenimages that represent the distribution of fluorophores for each of these regions are calculated. Then, based on the signal and noise boundaries established for each image patch, structural information is recovered. Each pixel is then divided into a subpixel grid and the coordinates of the emitters are estimated. (C) Finally, all the subpixel multiple signal classification (MUSIC) patches are stitched together and the super‐resolved image is formed. (D) Super‐resolved image of a synthetic nanoscopic structure with simulated blinking fluorophores placed along each ring^25^. Example generated using the MusiJ plugin for ImageJ with parameters: emission = 510 nm, numerical aperture = 1.49, magnification = 1, pixel size = 65 nm, threshold = −0.5, alpha = 2, subpixels per pixel = 5. Scale bars: 500 nm

Just a few months after SRRF was published back in 2016, the MUSICAL for FF‐SRM was released, presented as an ideal tool for live‐cell nanoscopy due to the minimum dataset size requirement: as few as 50 frames (Figure 8A) can provide a resolution enhancement, achieving ∼50 nm in live‐cell experiments. Because of the low number of frames required, MUSICAL can reconstruct super‐resolved images at a temporal spacing of less than 50 ms (with a maximum frame rate of 1000 fps) and represents a powerful option to study fast biological processes with nanoscopic resolution.^25^

Based on the MUSIC approach,^64^ MUSICAL relies on the singular value decomposition (SVD) principle. Briefly, SVD highlights the most prominent sources of variation within a system or a set of measurements by projecting the data onto a feature space.^65^ Unlike MUSIC, MUSICAL overcomes the need for impractically large datasets by applying a sliding soft window in which the MUSIC algorithm is implemented (Figure 8B). Given that the number of images required by MUSIC scales proportionally to the number of contrast sources (fluorophores) that are present, this sliding window feature restrains computational demands while providing accurate signal reconstructions in terms of resolution and image quality.^25^

First, once the sliding window function is defined and the image patches are created (Figure 8A), MUSIC calculates their eigenimages and their corresponding eigenvalues in order to recover the most prominent features of the sample by identifying the contrast sources within the signal (fluorescence; Figure 8B). Eigenimages are a collection of images that, summed together using their eigenvalue as weights, can be used to obtain the input image.^66^, ^67^, ^68^ The SVD principle also helps to suppress noise by filtering out those eigenimages with lower eigenvalues, for which a user‐defined threshold is used. Small (or near‐zero) eigenvalues are typically linked to a poor image signal and high noise. MUSICAL relies on the decomposition of the observation space (in this case, an image) into a source/signal (range) subspace and noise (null) subspace. This information helps the algorithm establish the boundaries of what is signal and what is background noise.

Next, structural information provided by MUSIC is enriched with prior knowledge of the PSF of the optical system, which provides regions of the likely position of the emitters by computing their projection in both the range and null The algorithm determines if the PSF is statistically represented by each subspace by analyzing their corresponding eigenvalues. To improve the estimation of the emitter location when the PSF calibration accuracy is low, a parameter *alpha* is used to narrow down the expected spread of the emitter. Typically, this parameter is chosen to be *alpha ≥ 2*. Finally, using the user‐defined parameter *sigma_0*, which serves as a threshold for the singular values of each eigenimage, the pixels in the image that correspond to the signal (range subspace) are separated from those that correspond to background noise (null subspace; Figure 8B). This last step generates the super‐resolved patches that are then stitched together to reconstruct the entire image (Figure 8C). An example of the processing of MUSICAL over simulated data is presented in Figure 8D.

Worth mentioning is that this patch (or subregion) analysis is different from that carried out by the parallelised version of 3B, which physically assigns each data patch to a specific core of the CPU (Central Processing Unit) (Figure 5D) for parallel processing, with the only aim to reduce the processing time. For the case of MUSICAL, the use of patch‐level analysis impacts directly the quality of the reconstruction since the MUSIC approach performs differently as a function of image size and emitter density.

MUSICAL does not require special blinking‐inducing photochemical treatment. It also performs relatively well in high‐density fluorophore conditions.^25^, ^69^ Unlike other methods such as STORM, which rely on long dark fluorophore states, MUSICAL performs well in both long‐ and short‐term dark state conditions. Additionally, despite blinking behaviour, the sliding window feature of MUSICAL allows the faithful reconstruction of the nanoscopic structures within the sample while maintaining the required dataset size small enough (a minimum of ∼50 frames).^25^ This facilitates the study of dynamic biological phenomena. Although less computationally demanding, MUSICAL performs poorly as the SNR diminishes.^70^ This condition becomes inconvenient in reduced light level experiments and makes this method prone to noise‐related artefact generation. Also on MUSICAL, both reconstruction ability and precision decrease proportionally with the sample brightness, which means that a poor photon emission will likely lead to false negatives in the resulting MUSICAL super‐resolution image. Increasing the laser power may help but it can lead to bleaching and phototoxicity. It is worth mentioning that MUSICAL requires a relatively accurate prior knowledge of the PSF of the microscope, which may otherwise lead to mild artefact generation. However, it behaves somewhat flexibly with respect to the estimation due to the *alpha* parameter mentioned above. The MUSICAL ImageJ plug‐in includes an automatic PSF estimation tool based on optical parameters (emission wavelength, NA, magnification and pixel size; code available at https://github.com/sebsacuna/MusiJ).

Similar to MUSICAL, another SRM method that provides resolution enhancement based on eigenimages calculation is SCORE (spatial covariance reconstructive microscopy).^71^ This method relies only on the information related to the range subspace, which limits its ability to reduce background‐induced artefacts. Also, SCORE lacks a sliding window function, which, in combination with a cost‐minimisation iteration‐like procedure, makes it a more computationally demanding method.

Overall, MUSICAL offers comparable and often superior results against most previously reported SRM methods, increasing resolution to below 50 nm. It performs competitively in a variety of experimental scenarios (such as fixed or live‐cell imaging and 3D nanoscopy) and also in terms of complexity, dataset size, computational times and highest resolution attainable. Its ability to reconstruct a super‐resolved image from a dataset of as small as ∼ 50 frames makes it a favoured choice for live‐cell nanoscopy. It is currently implemented in the MATLAB and FIJI/ImageJ platforms and does not require specialised instrumentation or complex sample treatment.^72^ However, reconstruction quality is severely affected by factors related to the overall quality of the signal, which includes fluorophore brightness, blinking dynamics and SNR.

## 7 MEAN‐SHIFT SUPER‐RESOLUTION (MSSR) MICROSCOPY

**FIGURE 9:**
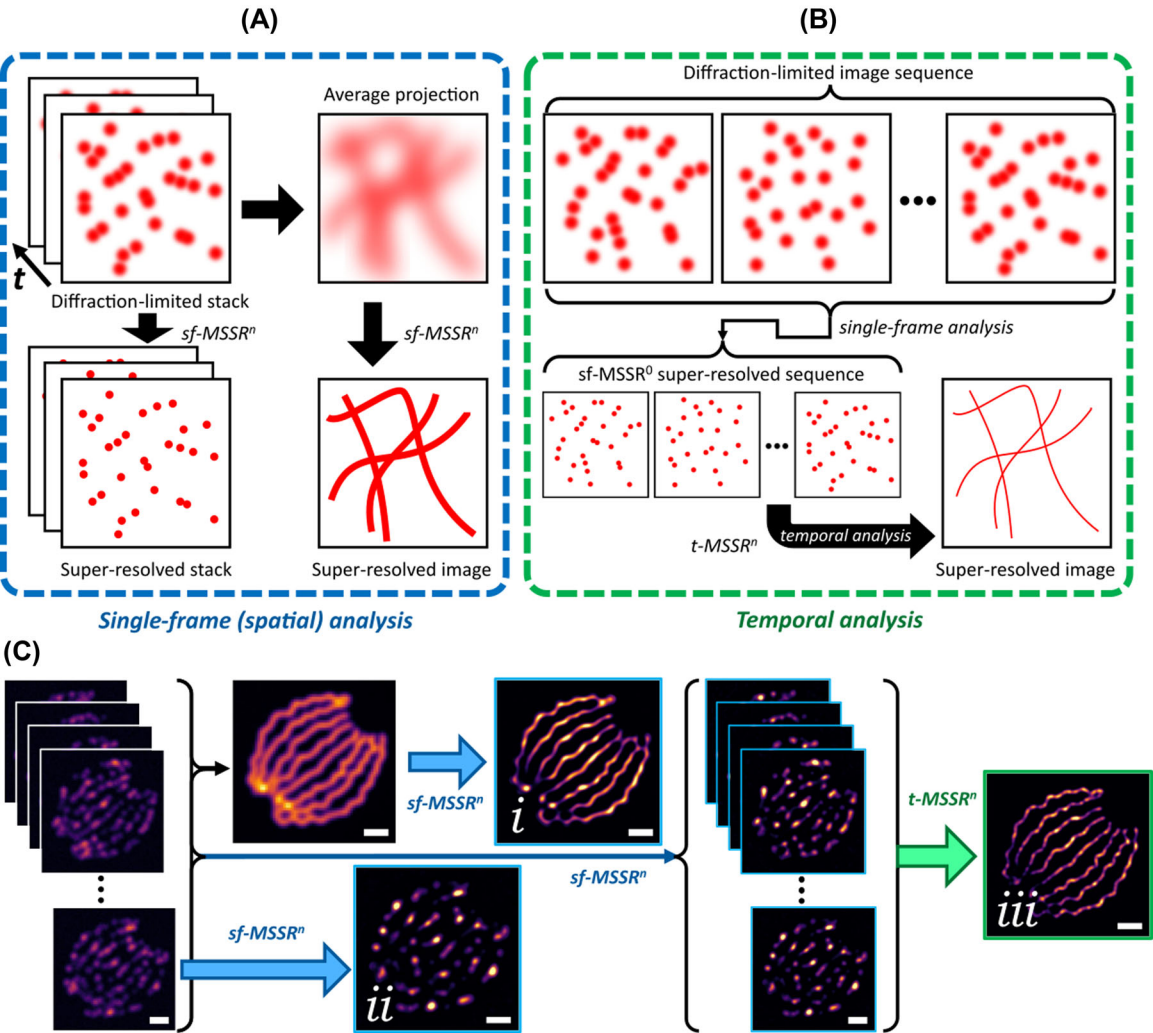
Mean‐shift super resolution (MSSR) microscopy. (A) Single‐frame MSSR (sf‐MSSR^n^) of a given order *n* reduces emitter width resulting in a super‐resolved image. This can be applied to either each image of a diffraction‐limited stack or to its average projection. (B) In MSSR temporal analysis (t‐MSSR^n^) the super‐resolved stack is obtained (a common step in both processing modalities) and then a given pixel‐wise temporal function is used to generate the reconstruction. (C) sf‐MSSR^n^ (i and ii) and t‐MSSR^n^ (iii) result from MSSR applied on a simulated microtubules dataset.^96^ Example generated using the MSSR plugin for ImageJ with parameters: Amp = 5, FWHM = 2, order = 1, interpolation = bicubic, meshing minimisation = enabled, temporal analysis = variance. Scale bars: 1 μm

The MSSR microscopy approach can obtain a super‐resolved image from a single diffraction‐limited image, achieving a resolution improvement of about a half (up to ∼140 nm; Figure 9A).^26^ MSSR is based on an idea similar to the radiality maps in SRRF. In addition, MSSR can be used as an FF‐SRM method by incorporating a temporal analysis, providing further spatial resolution enhancement and allowing the reliable discrimination of neighbouring emitters separated by 40 nm, by means of analyzing fewer than 30 frames (Figure 9B).

MSSR analysis shows striking denoising capabilities that outperform other FF‐SRM approaches, allowing the robust scrutiny of nanoscopic scales in a wide range of signal‐to‐noise ratio (SNR) conditions.^26^ MSSR is relatively robust in processing low‐ or high‐density fluorophore images and achieves comparable results in processing images collected with either CCD (Charge‐Coupled Device), sCMOS (scientific Complementary Metal‐Oxide‐Semiconductor) or photomultiplier‐based laser scanning technologies.

MSSR is based on MeanShift (MS) theory, which estimates local similarity properties between a central point and its neighbors.^73^, ^74^ MS is a vector that lies in the second‐order derivative space related to the data points and always points to the direction of the maximum local density of information. MS was conceptualised to iteratively climb through data points until reaching local density modes (stationary points for the iterative procedure) in the data space.^75^, ^76^ Most MS applications are distinguished by two main features: the search type for local density modes and the application of iterative procedures to find these modes.^76^, ^77^, ^78^, ^79^ These two features define the classical iterative procedure of MS. However, unlike the classical iterative procedure of MS, MSSR does not search modes along the data space and computes only the first MS value; this means that MSSR does not require MS iterations on its calculations.^26^

The single‐frame MSSR analysis (sf‐MSSR^n^; Figure 9A) is based on local kernel density estimation; hence, both spatial and range parameters are required.^26^ The spatial parameter is calculated from the optical properties of the imaging system and fluorophore features, namely, the pixel size of the diffraction‐limited image, the NA of the imaging lens and the emission wavelength of the fluorophore. The range parameter is defined automatically by the maximum difference of intensities in each neighbourhood that slides over the image.

MSSR offers the ability to select an iterative approach that provides higher spatial resolution (MSSR^n^).^26^ The authors refer to this as MSSR order, denoted by n. MSSR zero order (*n* = 0) is constituted by the computation of MS, reducing the FWHM of isolated emitters by about half. MSSR of higher orders (integer *n* > 0) is performed as an iterative procedure that applies basic algebraic functions, such as subtraction, multiplication, complement and normalisation, on consecutive resulting images, which reduces even further the FWHM of emitters. MSSR provides a further improvement in resolution as the order increases.

The sf‐MSSR^n^ resolution limit is reduced to 0.64 times the FWHM, and emitters at a smaller distance cannot be distinguished by sf‐MSSR analysis (for comparison, SRRF radiality map analysis provides an improvement in the resolution equivalent to 0.7 times the FWHM). In general, higher‐order MSSR analysis preserves the highest intensity of the original image but decreases the lower intensities progressively. For this reason, it is suitable to remove noise but harmful to the quality of the reconstructed image. Authors recommend using MSSR orders no greater than 3.

The MSSR temporal analysis (t‐MSSR^n^) integrates all the information over the sf‐MSSR^n^ super‐resolved stack by applying a pixel‐wise temporal function (PTF; Figure 9B). Each type of PTF has advantages depending on the nature of the fluorescence dynamics of the image stack to be processed. The t‐MSSR^n^ analysis achieves a higher resolution corresponding to 0.21 times the FWHM.

Since MSSR is not limited by detector architecture and can process both single images and image stacks, there are several scenarios of fluorescence microscopy and bioimaging where MSSR offers good performance (Figure 9C). Among other microscopy fields, sf‐MSSR^n^ is feasible in single‐particle tracking or fixed‐cell imaging microscopy, processing either a single image or each plane of a Z‐stack to obtain a 3D reconstruction. Given that sf‐MSSR^n^ has the property to operate over a single image, it is easily combinable with all FF‐SRM methods previously described to increase resolution on super‐resolved images. On the other hand, t‐MSSR^n^ is suitable for temporal multi‐frame analysis on fixed cells. In addition to the above, MSSR is compatible with other SRM techniques, such as SIM, STED, SOFI, 3B‐ODE, ESI, MUSICAL and SRRF, allowing a further resolution enhancement when applied to their super‐resolved images. This algorithm is available as a user‐friendly Fiji/ImageJ plug‐in.^26^

## 8 MACHINE LEARNING‐BASED FF‐SRM TECHNIQUES

**FIGURE 10:**
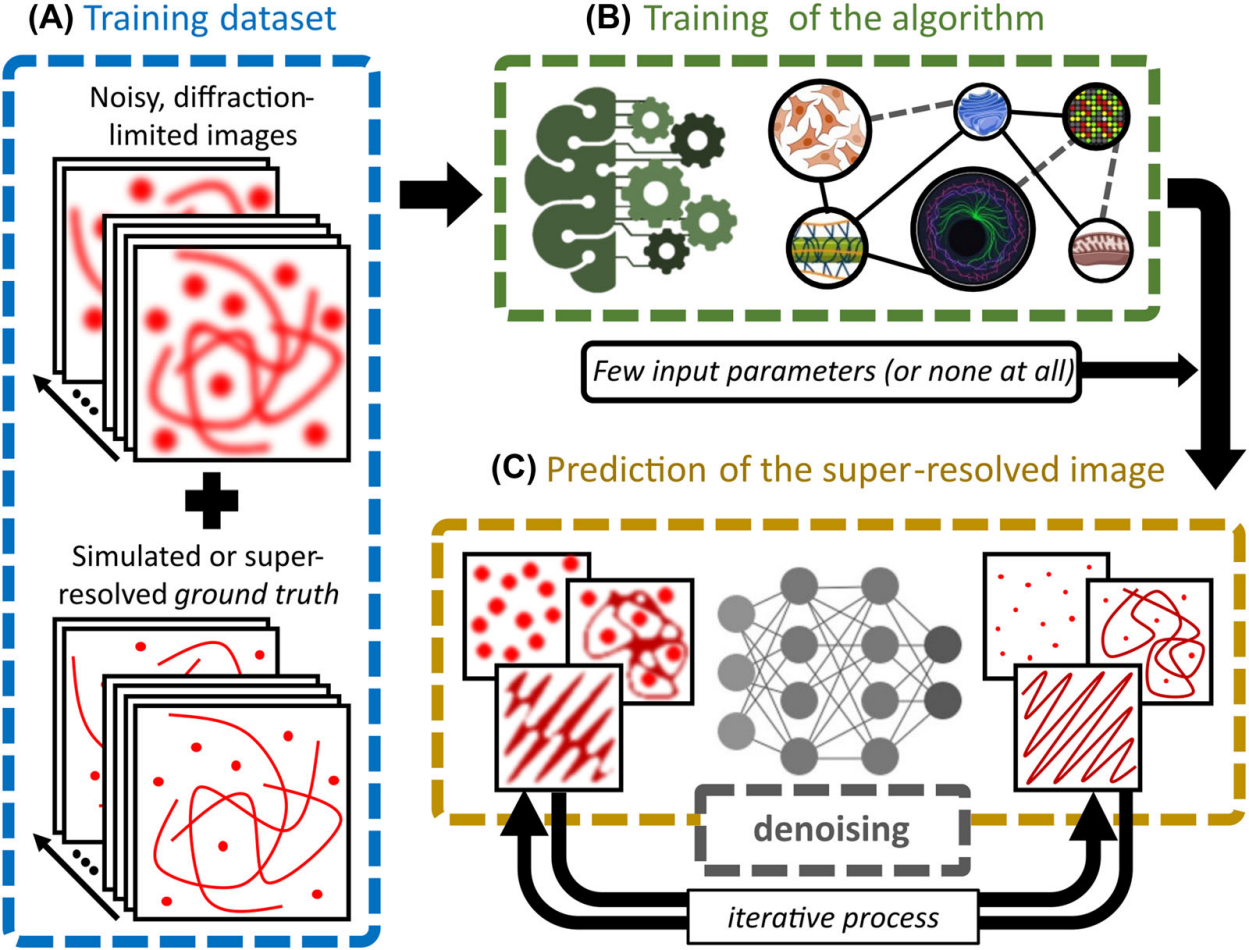
Deep‐learning super‐resolution. Most deep learning algorithms require training in the form of thousands of diffraction‐limited images and their corresponding expected results of enhanced resolution and contrast. These image pairs are commonly simulated but can also be acquired in a real optical system and super‐resolved with other SRM methods prior to training of the deep learning network. The demand of impractically large training datasets by deep learning approaches is compensated in the form of greatly improved computation times and almost parameter‐free operation. However, caution must be taken as reconstruction quality is highly dependent on training fitness

Deep learning encompasses a subset of machine learning algorithms, based on neural networks, which has gained popularity in recent years due to its great performance in different tasks such as segmentation,^80^ denoising,^81^ and SRM.^27^, ^82^, ^83^, ^84^, ^85^ In supervised deep learning, a training dataset, consisting of the input image and the expected result (ground truth), is used to tune the weights of a neural network. In the case of FF‐SRM, the training dataset encompasses a collection of image pairs, consisting of a low‐resolution (diffraction‐limited) image, and its corresponding SR image (the ground truth; Figure 10).

These approaches require a large number of images as training examples. For instance, deep learning‐guided Bayesian inference (DLBI) is trained with 12,000 examples,^27^ Deep‐Storm with 10,000 examples,^82^ and single‐frame super‐resolution microscopy (SFSRM) with 1000 examples.^84^ Such a large amount of training data is not always available to generate a good deep learning model, and there is not a general rule to decide the minimum number of images sufficient for the training.

DLBI^27^ is an algorithm that predicts an SRM image from a time series of high‐density fluorescent images. In the first step, the time series images are transformed into an SRM image using a deep learning architecture called generative adversarial network (GAN).^86^ The GAN is trained to simulate diffraction‐limited stacks from a high‐resolution image (12,000 images collected from Laplace‐filtered natural images and sketches). Therefore, to simulate the training dataset, DLBI encompasses an algorithm that, given a high‐resolution image, simulates the corresponding fluorescent time‐series images by means of using a Markov model that switches between emitting, not emitting and bleached states.

The algorithm takes into account expected photophysical properties of the emitters, such as switching probability between bright and dark excited states, as well as the PSF of the imaging system, in order to generate diffraction‐limited images very similar to those from microscope acquisitions. In a second step, the high‐resolution image predicted by the GAN is used to generate a set of possible fluorophore localisation. Bayesian inference with the time series images is used to discard a given localisation that is not well‐represented in the input dataset following a similar approach of 3B analysis.^22^

An advantage of DLBI, compared to 3B analysis, is that it reduces the computational time up to 100‐fold and generates comparable quality SRM images, scored by using the metrics of peak SNR, structural similarity and SQUIRREL analysis.^62^ Even though DLBI has advantages over 3B and the results look promising for a wide range of datasets, its widespread usage as FF‐SRM method is limited since there is not an easy‐to‐use distribution/program. The authors released a GitHub repository (https://github.com/liyu95/DLBI); however, its use requires advanced programming skills.

Sparsity‐based super‐resolution correlation microscopy (SPARCOM) is an FF‐SRM method that analyses a temporal sequence of diffraction‐limited images to model the behaviour of the temporal fluctuation of the fluorescence emitter, which creates an SRM image.^69^ SPARCOM is based on the theory of SMLM and aims to recover the position of the emitters in a finer grid. Using prior information about the PSF of the imaging system, SPARCOM formulates a multiple measurement vector (MMV) model to recover the position of the emitters and place its estimated variance value at the position.^87^ In SPARCOM, the MMV model includes the use of a covariance matrix for the optimisation of *N* ^4^ variables (unknowns). Taking into account the prior knowledge that emitters blink over time are uncorrelated over time and are sparse in the spatial domain, the number of unknown variables for the model is reduced to *N* ^2^, and these can be found using an iterative shrinkage‐thresholding algorithm (ISTA).^88^

SPARCOM can achieve a spatial resolution of ∼ 40 nm (similar to PALM and STORM) but through the analysis of only 50 frames. A disadvantage is the requirement of prior knowledge of the PSF and the need for the regularisation parameter ISTA (determined heuristically). The learned SPARCOM (LSPARCOM) approach encompasses an iterative algorithm that uses deep learning to predict super‐resolution images without the need of making any assumption about the PSF of the imaging system.^83^ It does not require fine‐tuning of the optimisation parameters, as with SPARCOM, and produces similar or better SRM images than SPARCOM using the SNR metric and by subjective visual comparison.

With LSPARCOM, the PSF and the regularisation parameter are learned from training data using a neural network approach (deep learning unfolding).^89^ The training dataset is generated by setting the position of the emitters (ground‐truth SRM image) from simulated biological microtubules or tubulins and then simulating the diffraction‐limited images using microscopy parameters such as sample thickness, random activation, laser power, lifetime of fluorophores, noise, PSF size, amongst others, in order to generate diffraction‐limited images very similar to those from microscope acquisitions. The training dataset allows LSPARCOM to map simulated diffraction‐limited images to SRM images since simulated images are similar to experimental images. Furthermore, it also allows the mapping of experimental diffraction‐limited images to the SRM reconstruction.

LSPARCOM generates SRM reconstructions similar to SPARCOM with a five‐fold computational time improvement.^83^ In addition, LSPARCOM can generate good quality SRM images with as few as 25 images. A disadvantage of the LSPARCOM approach is the requirement to test low‐resolution images to have a similar or not significantly different PSF to the images in the training dataset. If this is not the case, it can produce artefacts such as lines reconstructed as chains or intersection areas reconstructed as arcs. Similarly, LSPARCOM is sensitive to the pixel size of the simulated diffraction‐limited images used during training (a pixel size of 100 nm was used during training). If a significantly greater or lesser pixel size is used during the acquisition of the diffraction‐limited image, it can generate inaccurate SRM images. Therefore, in order to have good quality results on a dataset with different PSF or different pixel size than 100 nm, LSPARCOM must be retrained, which as stated previously is not an easy task. The authors provide a link to the full code and a graphic user interface.

Recently, a novel algorithm based on deep learning, SFSRM^84^ has been shown to achieve ∼20‐nm spatial resolution (a 10‐fold resolution improvement, compared to diffraction‐limited images). SFSRM uses two deep neural networks to obtain the SRM reconstruction. The first deep learning network, the signal‐enhancement network (SEN) receives as input the diffraction‐limited image (low‐SNR) and generates an image with high‐SNR (noise reduced) while maintaining the same resolution. The second deep learning network, super‐resolution network (SRN) receives as input the high‐SNR image and generates the SRM image. The training dataset for the SEN network (low‐SNR and high‐SNR) is obtained from fixed cells at different illumination intensities and from different microscopes (epifluorescence, TIRF, HILO [Highly inclined and laminated optical sheet] and confocal). This approach allows the network to reduce the noise in different microscopy techniques. The SRN network is then trained using simulated low‐resolution images (100‐nm pixel size) with their corresponding 10X high‐resolution image (10‐nm pixel size) and experimental diffraction‐limited wide‐field images with their high‐resolution images, which are reconstructed using STORM.^15^

The main feature of SFSRM is achieving ∼ 20 nm of resolution with a single diffraction‐limited image. SFSRM was used to visualise cargo transport dynamics in a dense microtubule network. The highest resolution reached by SFSRM is limited by the resolution from the training SR images (obtained with STORM); hence, the resolution can be increased if SRM with higher resolution is used for the training (e.g., MINFLUX microscope). The main disadvantage is that the SFSRM depends on the patterns observed during the training dataset. Therefore, it can produce erroneous SRM reconstructions for input images with different topological structures than those used in the training set (necessitating the retraining of the SFSRM network). Currently, there is no source code available to test SFSRM.

In summary, deep learning‐based algorithms are a powerful approach to obtain SR images since they are outperforming non‐machine learning‐based approaches in terms of computational time in generating an SR image, requiring fewer images to produce comparable SR images in terms of the resolution to non‐machine‐learning approaches, and even reaching resolutions of ∼ 20 nm within a single diffraction‐limited image. However, their utilisation is limited to a community with knowledge in machine learning due to the difficulty of optimising the parameters of the network, programming skills, a large number of training examples and high‐computational resources (access to GPU). There have been efforts to make deep learning algorithms available to novice users,^90^, ^91^, ^92^ for instance using ZeroCostDL4ML that provides easy‐to‐use Jupyter Notebooks for the training of deep neural networks in different tasks such as segmentation, denoising, object detection, super‐resolution and so forth. However, the only available method for super‐resolution is Deep‐STORM.^82^ The research community must continue this effort to make the algorithms easily available and user‐friendly since typically the end‐user does not have experience in machine learning to train or use deep learning networks.

## 9 SOFTWARE AVAILABILITY AND HOW TO CHOOSE THEM

Most of the methods presented in this work are implemented as free and open access software; however, their execution may require the use of multiple platforms such as Matlab, python, R, FIJI Plug‐in, amongst others.^21^, ^22^, ^23^, ^24^, ^25^, ^26^, ^27^ Due to the constant improvement in computing, memory and storage capacity, most modern computers meet the requirements to use the SRM software developed so far; however, a GPU is recommended to deploy its full potential, speeding up the data processing for certain software.

There is a wide range of bioimaging and fluorescence microscopy applications in which FF‐SRM methods can be used such as immunofluorescence of fixed cells, live‐cell imaging using organic dyes, fluorescent proteins or quantum dots, single‐particle tracking, colocalisation, 3D imaging and so forth.^21^, ^22^, ^23^, ^24^, ^25^, ^26^, ^27^ In the context of SRM, when it comes to choosing new approaches to analyse data, the highest attainable resolution and the number of images required to achieve such a goal are two closely related parameters that often lead to the selection of an approach over another.

Moreover, the nature of the input data themselves must be thrown into the equation to obtain the best results based on the available experimental conditions and resources. Methods such as SRRF, MUSICAL and MSSR are largely independent of fluorophore blinking dynamics and operate with relatively small dataset sizes, which makes these methods suitable for live‐cell imaging.^24^, ^25^, ^26^ On the other hand, software availability and ease of installation are factors that might ultimately define the course of action. While most SRM approaches are implemented on many of the most popular image analysis platforms, others might not be so readily reachable. Figure 3B and Table 1 show how all of the above‐mentioned aspects compare among the SRM methods discussed in this review, according to the experience of each author; therefore, we recommend identifying cases in which a particular FF‐SRM method has been used with similar purposes and experimental setups.

No matter which FF‐SRM method is chosen, we recommend characterising the fidelity of the SRM reconstruction with error mapping software like SQUIRREL^62^ or HAWKMAN.^93^ Computing global resolution indexes like resolution‐scaled Pearson, resolution‐scaled error^62^ or Fourier ring correlation^94^ are recommended to identify possible artefacts from loss of signal. With these metrics, a fair and systematic comparison between the FF‐SRM methods can be achieved and misinterpretation of the results or inappropriate implementation of the software can be diminished.

## 10 CONCLUDING REMARKS AND PERSPECTIVES

The development of FF‐SRM methods has facilitated the temporally resolved study of biology at the spatial nanoscale. Input images for most of these methods can be generated using microscopy platforms of general access, common fluorophores and simple sample preparation, making them suitable for use in most life science laboratories. That being said, it is nonetheless crucial to understand the basic concepts of these techniques and how each parameter will affect the final SRM reconstruction before choosing a particular FF‐SRM method.

No FF‐SRM technique is universally favoured in all experimental scenarios. As is so often the case with microscopy, high performance of a particular technique by one criterion (i.e., highest resolution possible) may come at the expense of poorer performance in others (i.e., susceptibility for generating artefacts, slow computational performance). While we have striven to indicate the general performance (and trade‐off) characteristics of the different approaches, there will always be scenarios in which any single algorithm may unexpectedly outperform another. This may be due to any combination of factors such as a previously untried application case and the shifting nature of bottle‐necks due to technological advancement over time. Due to the extensive circumstantial variables at play in each set of experiments, local factors will demand a degree of commitment to an empirical approach when choosing an FF‐SRM technique for any given analysis task.

In this regard, the diverse range of options for FF‐SRM mirrors the situation in the early (and still ongoing) years of the development of algorithms and implementations for SMLM image generation. A surge in interest in SMLM led to a rapid expansion of mathematical approaches for localising single‐molecule intensity fluctuations in temporal image series. Guidance for determining the strengths and weaknesses of the SMLM approaches advanced once canonical test data, simulated and real, were made available to the community and controlled criteria for assessing performance were applied in ‘challenges’. Most notably, the single‐molecule localisation challenge^95^ is available at https://srm.epfl.ch/. This ‘challenge’ approach has also been adopted in the 3D deconvolution microscopy challenge at http://bigwww.epfl.ch/deconvolution/challenge/index.html.

An equivalent ‘challenge’ for FF‐SRM is yet to be devised and is complicated by the wide flexibility and applicability of FF‐SRM techniques to a diverse range of microscopy modalities and experimental designs. A considerable task for setting up this endeavour will be to assemble a canonical dataset and universal performance criteria that will fairly and rigorously test the relative performance among the growing FF‐SRM family members. The value of such a ‘challenge’ is nevertheless unquestionable, in terms of time saved and errors avoided, for current and future researchers who seek to unlock the power of imaging to examine the most fundamental questions of biology at the highest spatial and temporal scales possible.

## AUTHOR CONTRIBUTIONS

Alma Alva, Eduardo Brito‐Alarcón and Adán Guerrero designed the review content: Introduction (Alma Alva, Eduardo Brito‐Alarcón, Esley Torres‐García), SOFI (Eduardo Brito‐Alarcón), 3B (Haydee O. Hernández), ESI (Damián Martínez) SRRF (Alma Alva), MUSICAL (Alejandro Linares), MSSR (Esley Torres‐García), machine learning (Paul Hernández‐Herrera), software availability (Raúl Pinto‐Cámara). Rocco D'Antuono, Christopher Wood and Adán Guerrero provided guidance, suggestions, concept and redaction validations. All the authors contributed to the conclusion section and approved the final version of the manuscript.

## References

[1] Jacquemet, G. , Carisey, A. F. , Hamidi, H. , Henriques, R. , & Leterrier, C. (2020). The cell biologist's guide to super‐resolution microscopy. Journal of Cell Science, 133(11), jcs240713. 32527967 10.1242/jcs.240713

[2] Lichtman, J. W. , & Conchello, J. A. (2005). Fluorescence microscopy. Nature Methods, 2(12), 910–919. 16299476 10.1038/nmeth817

[3] Vangindertael, J. , Camacho, R. , Sempels, W. , Mizuno, H. , Dedecker, P. , & Janssen, K. P. F. (2018). An introduction to optical super‐resolution microscopy for the adventurous biologist. Methods and Applications in Fluorescence, 6(2), 022003. 29422456 10.1088/2050-6120/aaae0c

[4] Schermelleh, L. , Ferrand, A. , Huser, T. , Eggeling, C. , Sauer, M. , Biehlmaier, O. , & Drummen, G P. C. (2019). Super‐resolution microscopy demystified. Nature Cell Biology, 21(1), 72–84. 30602772 10.1038/s41556-018-0251-8

[5] Rayleigh, L. (1903). On the theory of optical images, with special reference to the microscope. Journal of the Royal Microscopical Society, 23(4), 447–473.

[6] Hell, S. W. , & Wichmann, J. (1994). Breaking the diffraction resolution limit by stimulated emission: stimulated‐emission‐depletion fluorescence microscopy. Optics Letters, 19(11), 780. 19844443 10.1364/ol.19.000780

[7] Klar, T. A. , Jakobs, S. , Dyba, M. , Egner, A. , & Hell, S. W. (2000). Fluorescence microscopy with diffraction resolution barrier broken by stimulated emission. Proceedings of the National Academy of Sciences, 97(15), 8206–8210. 10.1073/pnas.97.15.8206 PMC26924 10899992

[8] Gustafsson, M. G. L. (2000). Surpassing the lateral resolution limit by a factor of two using structured illumination microscopy. SHORT COMMUNICATION. Journal of Microscopy, 198(2), 82–87. 10810003 10.1046/j.1365-2818.2000.00710.x

[9] Wegel, E. , Göhler, A. , Lagerholm, B. C. , Wainman, A. , Uphoff, S. , Kaufmann, R. , & Dobbie, I. M. (2016). Imaging cellular structures in super‐resolution with SIM, STED and Localisation Microscopy: A practical comparison. Scientific Reports, 6(1), 27290. 27264341 10.1038/srep27290 PMC4893670

[10] Butkevich, A. N. , Mitronova, G. Y. , Sidenstein, S. C. , Klocke, J. L. , Kamin, D. , Meineke, D. N. H. , D'este, E. , Kraemer, P. ‐T. , Danzl, J. G. , Belov, V. N. , & Hell, S. W. (2016). Fluorescent rhodamines and fluorogenic carbopyronines for super‐resolution STED microscopy in living cells. Angewandte Chemie International Edition, 55(10), 3290–3294. 26844929 10.1002/anie.201511018 PMC4770443

[11] Göttfert, F. , Wurm, C. A. , Mueller, V. , Berning, S. , Cordes, V. C. , Honigmann, A. , & Hell, S. W. (2013). Coaligned dual‐channel STED nanoscopy and molecular diffusion analysis at 20 nm resolution. Biophysical Journal, 105(1), L01–L03. 23823248 10.1016/j.bpj.2013.05.029 PMC3699760

[12] Hofmann, M. , Eggeling, C. , Jakobs, S. , & Hell, S. W. (2005). Breaking the diffraction barrier in fluorescence microscopy at low light intensities by using reversibly photoswitchable proteins. Proceedings of the National Academy of Sciences, 102(49), 17565–17569. 10.1073/pnas.0506010102 PMC1308899 16314572

[13] Balzarotti, F. , Eilers, Y. , Gwosch, K. C. , Gynnå, A. H. , Westphal, V. , Stefani, F. D. , Elf, J. , & Hell, S. W. (2017). Nanometer resolution imaging and tracking of fluorescent molecules with minimal photon fluxes. Science, 355(6325), 606–612. 28008086 10.1126/science.aak9913

[14] Lelek, M. L. , Gyparaki, M. T. , Beliu, G. , Schueder, F. , Griffié, J. , Manley, S. , Jungmann, R. , Sauer, M. , Lakadamyali, M. , & Zimmer, C. (2021). Single‐molecule localization microscopy. Nature Reviews Methods Primers, 1(1), 39. 10.1038/s43586-021-00038-x PMC9160414 35663461

[15] Rust, M. J. , Bates, M. , & Zhuang, X. (2006). Sub‐diffraction‐limit imaging by stochastic optical reconstruction microscopy (STORM). Nature Methods, 3(10), 793–796. 16896339 10.1038/nmeth929 PMC2700296

[16] Schnitzbauer, J. , Strauss, M. T. , Schlichthaerle, T. , Schueder, F. , & Jungmann, R. (2017). Super‐resolution microscopy with DNA‐PAINT. Nature Protocols, 12(6), 1198–1228. 28518172 10.1038/nprot.2017.024

[17] Sharonov, A. , & Hochstrasser, R. M. (2006). Wide‐field subdiffraction imaging by accumulated binding of diffusing probes. Proceedings of the National Academy of Sciences, 103(50), 18911–18916. 10.1073/pnas.0609643104 PMC1748151 17142314

[18] Betzig, E. , Patterson, G. H. , Sougrat, R. , Lindwasser, O. W. , Olenych, S. , Bonifacino, J S. , Davidson, M W. , Lippincott‐Schwartz, J. , & Hess, H. F. (2006). Imaging Intracellular Fluorescent Proteins at Nanometer Resolution. Science, 313(5793), 1642–1645. 16902090 10.1126/science.1127344

[19] Minoshima, M. , & Kikuchi, K. (2017). Photostable and photoswitching fluorescent dyes for super‐resolution imaging. JBIC Journal of Biological Inorganic Chemistry, 22(5), 639–652. 28083655 10.1007/s00775-016-1435-y

[20] Lin, Y. , Long, J. J. , Huang, F. , Duim, W. C. , Kirschbaum, S. , Zhang, Y. , Schroeder, L. K. , Rebane, A. A. , Velasco, M. G. M. , Virrueta, A. , Moonan, D. W. , Jiao, J. , Hernandez, S. Y. , Zhang, Y. , & Bewersdorf, J. (2015). Quantifying and optimizing single‐molecule switching nanoscopy at high speeds. PLoS ONE, 10(5), e0128135. 26011109 10.1371/journal.pone.0128135 PMC4444241

[21] Dertinger, T. , Colyer, R. , Iyer, G. , Weiss, S. , & Enderlein, J. (2009). Fast, background‐free, 3D super‐resolution optical fluctuation imaging (SOFI). Proceedings of the National Academy of Sciences, 106(52), 22287–22292. 10.1073/pnas.0907866106 PMC2799731 20018714

[22] Cox, S. , Rosten, E. , Monypenny, J. , Jovanovic‐Talisman, T. , Burnette, D. T. , Lippincott‐Schwartz, J. , Jones, G. E. , & Heintzmann, R. (2011). Bayesian localization microscopy reveals nanoscale podosome dynamics. Nature Methods, 9(2), 195–200. 22138825 10.1038/nmeth.1812 PMC3272474

[23] Yahiatene, I. , Hennig, S. , Müller, M. , & Huser, T. (2015). Entropy‐based super‐resolution imaging (ESI): From disorder to fine detail. ACS Photonics, 2(8), 1049–1056.

[24] Gustafsson, N. , Culley, S. N. , Ashdown, G. , Owen, D. M. , Pereira, P. M. , & Henriques, R. (2016). Fast live‐cell conventional fluorophore nanoscopy with ImageJ through super‐resolution radial fluctuations. Nature Communications, 7(1), 12471. 10.1038/ncomms12471 PMC4990649 27514992

[25] Agarwal, K. , & Macháň, R. (2016). Multiple signal classification algorithm for super‐resolution fluorescence microscopy. Nature Communications, 7(1), 13752. 10.1038/ncomms13752 PMC5155148 27934858

[26] García, E. T. , Cámara, R. P. , Linares, A. , Martínez, D. , Abonza, V. , Brito‐Alarcon, E. , Calcines‐Cruz, C. , Galindo, G. , Torres, D. , Jabloñski, M. , Torres‐Martínez, H. H. , Martínez, J. , Hernández, H. , Ocelotl, J. , Garcés, Y. , Barchi, M. , Dubrovsky, J. G. , Darszon, A. , Buffone, M. , … Guerrero, A. (2021). Nanoscopic resolution within a single imaging frame. bioRxiv., 2021.10.17.464398.

[27] Li, Y. , Xu, F. , Zhang, F. , Xu, P. , Zhang, M. , Fan, M. , Li, L. , Gao, X. , & Han, R. (2018). DLBI: Deep learning guided Bayesian inference for structure reconstruction of super‐resolution fluorescence microscopy. Bioinformatics, 34(13), i284–i294. 29950012 10.1093/bioinformatics/bty241 PMC6022599

[28] Moeyaert, B. , Vandenberg, W. , & Dedecker, P. (2020). SOFIevaluator: A strategy for the quantitative quality assessment of SOFI data. Biomedical Optics Express, 11(2), 636. 32133218 10.1364/BOE.382278 PMC7041449

[29] Geissbuehler, S. , Dellagiacoma, C. , & Lasser, T. (2011). Comparison between SOFI and STORM. Biomedical Optics Express, 2(3), 408–420. 21412447 10.1364/BOE.2.000408 PMC3047347

[30] Dertinger, T. , Colyer, R. , Vogel, R. , Unity Health Toronto, J. R. , & Weiss, S. (2010). Achieving increased resolution and more pixels with Superresolution *Optica*l Fluctuation Imaging (SOFI). Optics Express, 18(18), 18875–18885. 20940780 10.1364/OE.18.018875 PMC3072111

[31] Mendel, J. M. (1991). Tutorial on higher‐order statistics (spectra) in signal processing and system theory: Theoretical results and some applications. Proceedings of the IEEE, 79(3), 278–305.

[32] Pawlowska, M. , Tenne, R. , Ghosh, B. , Makowski, A. , & Lapkiewicz, R. (2022). Embracing the uncertainty: The evolution of SOFI into a diverse family of fluctuation‐based super‐resolution microscopy methods. Journal of Physics: Photonics, 4(1), 012002.

[33] Sibarita, J. ‐B. (2005). Deconvolution microscopy. Advances in Biochemical Engineering/Biotechnology, 95, 201–243. 16080270 10.1007/b102215

[34] Dertinger, T. , Xu, J. , Naini, O. , Vogel, R. , & Weiss, S. (2012). SOFI‐based 3D superresolution sectioning with a widefield microscope. Optical Nanoscopy, 1(1), 2. 24163789 10.1186/2192-2853-1-2 PMC3807127

[35] Gallina, M. E. , Xu, J. , Dertinger, T. , Aizer, A. , Shav‐Tal, Y. , & Weiss, S. (2013). Resolving the spatial relationship between intracellular components by dual color super resolution optical fluctuations imaging (SOFI)." Optical Nanoscopy, 2(1), 2. 10.1186/2192-2853-2-2 PMC3855418 24324919

[36] Geissbueler, S. , Bocchio, N. L. , Dellagiacoma, C. , Berclaz, C. , Leutenegger, M. , & Lasser, T. (2012). Mapping molecular statistics with balanced super‐resolution optical fluctuation imaging (bSOFI). Optical Nanoscopy, 1(1), 4.

[37] Geissbuehler, S. , Sharipov, A. , Godinat, A. L. , Bocchio, N. L. , Sandoz, P. A. , Huss, A. , Jensen, N. A. , Jakobs, S. , Enderlein, J. R. , Gisou Van Der Goot, F. , Dubikovskaya, E. A. , Lasser, T. , & Leutenegger, M. (2014). Live‐cell multiplane three‐dimensional super‐resolution optical fluctuation imaging. Nature Communications, 5(1), 5830. 10.1038/ncomms6830 PMC4284648 25518894

[38] Deschout, H. , Lukes, T. , Sharipov, A. , Szlag, D. , Feletti, L. , Vandenberg, W. , Dedecker, P. , Hofkens, J. , Leutenegger, M. , Lasser, T. , & Radenovic, A. (2016). Complementarity of PALM and SOFI for super‐resolution live‐cell imaging of focal adhesions. Nature Communications, 7(1), 13693. 10.1038/ncomms13693 PMC5187410 27991512

[39] Kodama, Y. , & Hu, C. ‐D. (2012). Bimolecular fluorescence complementation (BiFC): A 5‐year update and future perspectives. BioTechniques, 53(5), 285–298. 23148879 10.2144/000113943

[40] Hertel, F. , Mo, G. C. H. , Duwé, S. , Dedecker, P. , & Zhang, J. (2016). RefSOFI for mapping nanoscale organization of protein‐protein interactions in living cells. Cell Reports, 14(2), 390–400. 26748717 10.1016/j.celrep.2015.12.036 PMC4870019

[41] Yi, X. , & Weiss, S. (2020). Cusp‐artifacts in high order superresolution optical fluctuation imaging. Biomedical Optics Express, 11(2), 554. 32206387 10.1364/BOE.382296 PMC7041480

[42] Yi, X. , Son, S. , Ando, R. , Miyawaki, A. , & Weiss, S. (2019). Moments reconstruction and local dynamic range compression of high order superresolution optical fluctuation imaging. Biomedical Optics Express, 10(5), 2430. 31149378 10.1364/BOE.10.002430 PMC6524576

[43] Vandenberg, W. , Leutenegger, M. , Duwé, S. , & Dedecker, P. (2019). An extended quantitative model for super‐resolution optical fluctuation imaging (SOFI). Optics Express, 27(18), 25749. 31510441 10.1364/OE.27.025749

[44] Dedecker, P. , Duwé, S. , Neely, R. K. , & Zhang, J. (2012). Localizer: Fast, accurate, open‐source, and modular software package for superresolution microscopy. Journal of Biomedical Optics, 17(12), 126008–126008. 23208219 10.1117/1.JBO.17.12.126008 PMC3512108

[45] Miao, Y. , Weiss, S. , & Yi, X. (2022). PySOFI: An open source Python package for SOFI. Biophysical Reports, 2(2), 100052. 36425773 10.1016/j.bpr.2022.100052 PMC9680711

[46] Xu, F. , Zhang, M. , Liu, Z. , Xu, P. , & Zhang, F. (2015). Bayesian localization microscopy based on intensity distribution of fluorophores. Protein & Cell, 6(3), 211–220. 25672498 10.1007/s13238-015-0133-9 PMC4348249

[47] Rosten, E. , Jones, G. E. , & Cox, S. (2013). ImageJ plug‐in for Bayesian analysis of blinking and bleaching. Nature Methods, 10(2), 97–98. 23361088 10.1038/nmeth.2342

[48] Hu, Y. S. , Nan, X. , Sengupta, P. , Lippincott‐Schwartz, J. , & Cang, H. (2013). Accelerating 3B single‐molecule super‐resolution microscopy with cloud computing. Nature Methods, 10(2), 96–97. 23361087 10.1038/nmeth.2335 PMC4235519

[49] Hernández, H. O. , Hidalgo, P. , Wood, C. D. , González, R. , & Guerrero, A. (2016). High performance computer applications. Communications in Computer and Information Science, 595, 356–366.

[50] Xu, F. , Zhang, M. , He, W. , Han, R. , Xue, F. , Liu, Z. , Zhang, F. , Lippincott‐Schwartz, J. , & Xu, P. (2017). Live cell single molecule‐guided Bayesian localization super resolution microscopy. Cell Research, 27(5), 713–716. 28035138 10.1038/cr.2016.160 PMC5520847

[51] Li, H. , Xu, F. , Gao, S. , Zhang, M. , Xue, F. , Xu, P. , & Zhang, F. (2020). Live‐SIMBA: An ImageJ plug‐in for the universal and accelerated single molecule‐guided Bayesian localization super resolution microscopy (SIMBA) method. Biomedical Optics Express, 11(10), 5842. 33149990 10.1364/BOE.404820 PMC7587271

[52] Suárez, Y. G. , Martínez, J. L. , Hernández, D. T. , Hernández, H. O. , Hernández, H. O. , Pérez‐Delgado, A. , Méndez, M. , Wood, C. D. , Rendon‐Mancha, J. M. , Silva‐Ayala, D. , López, L. , Guerrero, A. , & Arias, C. F. (2019). Nanoscale organization of rotavirus replication machineries. eLife, 8, e42906. 31343403 10.7554/eLife.42906 PMC6692110

[53] Fazel, M. , Wester, M. J. , Mazloom‐Farsibaf, H. , Meddens, M. B. M. , Eklund, A. S. , Schlichthaerle, T. , Schueder, F. , Jungmann, R. , & Lidke, K. A. (2019). Bayesian multiple emitter fitting using reversible jump Markov chain Monte Carlo. Scientific Reports, 9(1), 13791. 31551452 10.1038/s41598-019-50232-x PMC6760159

[54] Chen, R. , Zhao, Y. , Li, M. , Wang, Y. , Zhang, L. , & Fei, P. (2020). Efficient superâ resolution volumetric imaging by radial fluctuation Bayesian analysis lightâ sheet microscopy. Journal of Biophotonics, 13(8), e201960242. 32314491 10.1002/jbio.201960242

[55] Diekmann, R. , Helle, Ø. I. , Øie, C. I. , Mccourt, P. , Huser, T. R. , Schüttpelz, M. , & Ahluwalia, B. S. (2017). Chip‐based wide field‐of‐view nanoscopy. Nature Photonics, 11(5), 322–328.

[56] Culley, S. , Tosheva, K. L. , Pereira, P. M. , & Henriques, R. (2018). SRRF: Universal live‐cell super‐resolution microscopy. The International Journal of Biochemistry & Cell Biology, 101, 74–79. 29852248 10.1016/j.biocel.2018.05.014 PMC6025290

[57] Stubb, A. , Laine, R. F. , Miihkinen, M. , Hamidi, H. , Guzmán, C. , Henriques, R. , Jacquemet, G. , & Ivaska, J. (2020). Fluctuation‐based super‐resolution traction force microscopy. Nano Letters, 20(4), 2230–2245. 32142297 10.1021/acs.nanolett.9b04083 PMC7146861

[58] Yao, L. , Zhang, L. , Chen, L. , Gong, X. , Zhong, J. , Wang, B. , Fei, Y. , Mi, L. , & Ma, J. (2021). Dynamic structure of yeast septin by fast fluctuation‐enhanced structured illumination microscopy. Microorganisms, 9(11), 2255. 34835381 10.3390/microorganisms9112255 PMC8620077

[59] Han, Y. , Lu, X. , Zhang, Z. , Liu, W. , Chen, Y. , Liu, X. , Hao, X. , & Kuang, C. (2019). Ultra‐fast, universal super‐resolution radial fluctuations (SRRF) algorithm for live‐cell super‐resolution microscopy. Optics Express, 27(26), 38337. 31878602 10.1364/OE.27.038337

[60] Wang, B. , Yao, L. , Jing, Y. , Fei, Y. , Bai, Q. , Mi, L. , & Ma, J. (2020). Multicomposite superâ resolution microscopy: Enhanced Airyscan resolution with radial fluctuation and sample expansions. Journal of Biophotonics, 13(5), e2419. 31999066 10.1002/jbio.201960211

[61] Yi, X. , Son, S. , Ando, R. , Miyawaki, A. , & Weiss, S. (2019). Moments reconstruction and local dynamic range compression of high order superresolution optical fluctuation imaging. Biomedical Optics Express, 10(5), 2430. 31149378 10.1364/BOE.10.002430 PMC6524576

[62] Culley, S. N. , Albrecht, D. , Jacobs, C. , Pereira, P. M. , Leterrier, C. , Mercer, J. , & Henriques, R. (2018). Quantitative mapping and minimization of super‐resolution optical imaging artifacts. Nature Methods, 15(4), 263–266. 29457791 10.1038/nmeth.4605 PMC5884429

[63] Laine, R. F. , Heil, H. S. , Coelho, S. , Nixon‐Abell, J. , Jimenez, A. , Galgani, T. , Stubb, K. , Follian, G. , Culley, S. , Jacquemet, G. , Hajj, B. , Leterrier, C. , & Henriques, R. (2022). High‐fidelity 3D live‐cell nanoscopy through data‐driven enhanced super‐resolution radial fluctuation. BioRxiv, 2022.04.07.487490. 10.1038/s41592-023-02057-w PMC10703683 37957430

[64] Schmidt, R. (1986). Multiple emitter location and signal parameter estimation. IEEE Transactions on Antennas and Propagation, 34(3), 276–280.

[65] Stewart, G. W. (1993). On the early history of the singular value decomposition. SIAM Review, 35(4), 551–566.

[66] Turk, M. , & Pentland, A. (1991). Eigenfaces for recognition. Journal of Cognitive Neuroscience, . 10.1162/jocn.1991.3.1.71 23964806

[67] Leonardis, A. , & Bischof, H. (2000). Robust recognition using eigenimages. Computer Vision and Image Understanding, 78(1), 99–118.

[68] Monwar, M. M. , Rezaei, S. , & Prkachin, K. (2007). Eigenimage based pain expression recognition. International Journal of Applied Mathematics, 36(2), 1–6.

[69] Solomon, O. , Mutzafi, M. , Segev, M. , & Eldar, Y. C. (2018). Sparsity‐based super‐resolution microscopy from correlation information. Optics Express, 26(14), 18238. 30114104 10.1364/OE.26.018238

[70] Opstad, I. S. , Acuña, S. , Hernandez, L. E. V. , Cauzzo, J. , Škalko‐Basnet, N. , Ahluwalia, B. S. , & Agarwal, K. (2020). Fluorescence fluctuations‐based super‐resolution microscopy techniques: an experimental comparative study. arXiv, .

[71] Deng, Y. , Sun, M. , Lin, P. ‐H. , Ma, J. , & Shaevitz, J. W. (2014). Spatial covariance reconstructive (SCORE) super‐resolution fluorescence microscopy. PLoS ONE, 9(4), e94807. 24788039 10.1371/journal.pone.0094807 PMC4005777

[72] Acuña, S. , Ströhl, F. , Opstad, I. S. , Ahluwalia, B. S. , & Agarwal, K. (2020). MusiJ: An ImageJ plugin for video nanoscopy. Biomedical Optics Express, 11(5), 2548. 32499942 10.1364/BOE.382735 PMC7249817

[73] Fukunaga, K. , & Hostetler, L. (1975). The estimation of the gradient of a density function, with applications in pattern recognition. IEEE Transactions on Information Theory, 21(1), 32–40.

[74] Yizong, C. (1995). Mean shift, mode seeking, and clustering. IEEE Transactions on Pattern Analysis and Machine Intelligence, 17(8), 790–799.

[75] Rao, S. , De Medeiros Martins, A. , & Prã­Ncipe, J. C. (2009). Mean shift: An information theoretic perspective. Pattern Recognition Letters, 30(3), 222–230.

[76] Comaniciu, D. , & Meer, P. (2002). Mean shift: A robust approach toward feature space analysis. IEEE Transactions on Pattern Analysis and Machine Intelligence, 24(5), 603–619.

[77] Hu, J. ‐S. , Juan, C. ‐W. , & Wang, J. ‐J. (2008). A spatial‐color mean‐shift object tracking algorithm with scale and orientation estimation. Pattern Recognition Letters, 29(16), 2165–2173.

[78] Fazekas, F. J. , Shaw, T. R. , Kim, S. , Bogucki, R. A. , & Veatch, S. L. (2021). A mean shift algorithm for drift correction in localization microscopy. Biophysical Reports, 1(1), 100008. 35382035 10.1016/j.bpr.2021.100008 PMC8978553

[79] Barash, D. , & Comaniciu, D. (2004). A common framework for nonlinear diffusion, adaptive smoothing, bilateral filtering and mean shift. Image and Vision Computing, 22(1), 73–81.

[80] Ronneberger, O. , Fischer, P. , & Brox, T. (2015). U‐Net: Convolutional Networks for Biomedical Image Segmentation. Medical Image Computing and Computer‐Assisted Intervention–MICCAI 2015, 18th International Conference, Munich, Germany, October 5‐9, 2015, Proceedings, Part III." Lecture Notes in Computer Science, (pp. 234–241). Springer.

[81] Krull, A. , Buchholz, T. ‐O. , & Jug, F. (2019). Noise2Void–Learning denoising from single noisy images. 2019 IEEE/CVF Conference on Computer Vision and Pattern Recognition (CVPR), Long Beach, CA (pp. 2124–2132).

[82] Nehme, E. , Weiss, L. E. , Michaeli, T. , & Shechtman, Y. (2018). Deep‐STORM: Super‐resolution single‐molecule microscopy by deep learning. Optica, 5(4), 458.

[83] Dardikman‐Yoffe, G. , & Eldar, Y. C. (2020). Learned SPARCOM: Unfolded deep super‐resolution microscopy. Optics Express, 28(19), 27736. 32988061 10.1364/OE.401925

[84] Chen, R. , Tang, X. , Shen, Z. , Shen, Y. , Tiantian, L. , Ji, W. , & Yao, S. (2021). Deep‐learning super‐resolution microscopy reveals nanometer‐scale intracellular dynamics at the millisecond temporal resolution. bioRxiv, 2021.10.08.463746.

[85] Ouyang, W. , Aristov, A. , Lelek, M. L. , Hao, X. , & Zimmer, C. (2018). Deep learning massively accelerates super‐resolution localization microscopy. Nature Biotechnology, 36(5), 460–468. 10.1038/nbt.4106 29658943

[86] Goodfellow, I. J. , Pouget‐Abadie, J. , Mirza, M. , Xu, B. , Warde‐Farley, D. , Ozair, S. , Courville, A. , & Bengio, Y. (2014). Generative adversarial networks. arXiv, .

[87] Davies, M. E. , & Eldar, Y. C. (2012). Rank awareness in joint sparse recovery. IEEE Transactions on Information Theory, 58(2), 1135–1146.

[88] Rozell, C. J. , Johnson, D. H. , Baraniuk, R. G. , & Olshausen, B. A. (2008). Sparse coding via thresholding and local competition in neural circuits. Neural Computation, 20(10), 2526–2563. 18439138 10.1162/neco.2008.03-07-486

[89] Gregor, K. , & LeCun, Y. (2021). Learning fast approximations of sparse coding. Proceedings of the 27th International Conference on International Conference on Machine Learning (ICML’10), Haifa, Israel.

[90] Von Chamier, L. , Laine, R. F. , Jukkala, J. , Spahn, C. , Krentzel, D. , Nehme, E. , Lerche, M. , Hernández‐Pérez, S. , Mattila, P. K. , Karinou, E. , Holden, S. , Solak, A. C. , Krull, A. , Buchholz, T. ‐O. , Jones, M. L. , Royer, L. C. A. , Leterrier, C. , Shechtman, Y. , Jug, F. , … Henriques, R. (2021). Democratising deep learning for microscopy with ZeroCostDL4Mic. Nature Communications, 12(1), 2276. 10.1038/s41467-021-22518-0 PMC8050272 33859193

[91] Gómez ‐De‐Mariscal, E. , García‐López‐De‐Haro, C. , Ouyang, W. , Donati, L. , Lundberg, E. , Unser, M. , Muñoz‐Barrutia, A. , & Sage, D. (2021). DeepImageJ: A user‐friendly environment to run deep learning models in ImageJ. Nature Methods, 18(10), 1192–1195. 34594030 10.1038/s41592-021-01262-9

[92] Körber, N. (2022). MIA: An open source standalone deep learning application for microscopic image analysis. bioRxiv, 2022.01.14.476308. 10.1016/j.crmeth.2023.100517 PMC10391334 37533647

[93] Marsh, R. J. , Costello, I. , Gorey, M. ‐A. , Ma, D. , Huang, F. , Gautel, M. , Parons, M. , & Cox, S. (2021). Sub‐diffraction error mapping for localisation microscopy images. Nature Communications, 12(1), 5611. 10.1038/s41467-021-25812-z PMC8460687 34556647

[94] Banterle, N. , Bui, K. H. , Lemke, E. A. , & Beck, M. (2013). Fourier ring correlation as a resolution criterion for super‐resolution microscopy. Journal of Structural Biology, 183(3), 363–367. 23684965 10.1016/j.jsb.2013.05.004

[95] Sage, D. , Pham, T. ‐A. , Babcock, H. , Lukes, T. , Pengo, T. , Chao, J. , Velmurugan, R. , Herbert, A. , Agrawal, A. , Colabrese, S. , Wheeler, A. , Archetti, A. , Rieger, B. , Ober, R. , Hagen, G M. , Sibarita, J. ‐B. , Ries, J. , Henriques, R. , Unser, M. , & Holden, S. (2019). Super‐resolution fight club: assessment of 2D and 3D single‐molecule localization microscopy software. Nature Methods, 16(5), 387–395. 30962624 10.1038/s41592-019-0364-4 PMC6684258

[96] Sage, D. , Kirshner, H. , Pengo, T. , Stuurman, N. , Min, J. , Manley, S. , & Unser, M. (2015). Quantitative evaluation of software packages for single‐molecule localization microscopy. Nature Methods, 12(8), 717–724. 26076424 10.1038/nmeth.3442

